# Interactions Between Adiponectin-Pathway Polymorphisms and Obesity on Postmenopausal Breast Cancer Risk Among African American Women: The WHI SHARe Study

**DOI:** 10.3389/fonc.2021.698198

**Published:** 2021-07-21

**Authors:** Gina E. Nam, Zuo-Feng Zhang, Jianyu Rao, Hua Zhou, Su Yon Jung

**Affiliations:** ^1^ Department of Epidemiology, Fielding School of Public Health, University of California at Los Angeles (UCLA), Los Angeles, CA, United States; ^2^ Center for Human Nutrition, Department of Medicine, UCLA David Geffen School of Medicine, University of California at Los Angeles (UCLA), Los Angeles, CA, United States; ^3^ Department of Biostatistics, Fielding School of Public Health, University of California at Los Angeles (UCLA), Los Angeles, CA, United States; ^4^ Translational Sciences Section, School of Nursing, University of California at Los Angeles (UCLA), Los Angeles, CA, United States; ^5^ Jonsson Comprehensive Cancer Center, University of California at Los Angeles (UCLA), Los Angeles, CA, United States

**Keywords:** obesity, adiponectin, postmenopausal breast cancer, African American women, single nucleotide polymorphism

## Abstract

**Background:**

A decreased level of serum adiponectin is associated with obesity and an increased risk of breast cancer among postmenopausal women. Yet, the interplay between genetic variants associated with adiponectin phenotype, obesity, and breast cancer risk is unclear in African American (AA) women.

**Methods:**

We examined 32 single-nucleotide polymorphisms (SNPs) previously identified in genome-wide association and replication studies of serum adiponectin levels using data from 7,991 AA postmenopausal women in the Women’s Health Initiative SNP Health Association Resource.

**Results:**

Stratifying by obesity status, we identified 18 adiponectin-related SNPs that were associated with breast cancer risk. Among women with BMI ≥ 30 kg/m^2^, the minor TT genotype of *FER* rs10447248 had an elevated breast cancer risk. Interaction was observed between obesity and the CT genotype of *ADIPOQ* rs6773957 on the additive scale for breast cancer risk (relative excess risk due to interaction, 0.62; 95% CI, 0.32–0.92). The joint effect of BMI ≥ 30 kg/m^2^ and the TC genotype of *OR8S1* rs11168618 was larger than the sum of the independent effects on breast cancer risk.

**Conclusions:**

We demonstrated that obesity plays a significant role as an effect modifier in an increased effect of the SNPs on breast cancer risk using one of the most extensive data on postmenopausal AA women.

**Impact:**

The results suggest the potential use of adiponectin genetic variants as obesity-associated biomarkers for informing AA women who are at greater risk for breast cancer and also for promoting behavioral interventions, such as weight control, to those with risk genotypes.

## Introduction

Obesity, defined as body mass index (BMI) of 30.0 kg/m^2^ or greater, is a well-established risk factor for postmenopausal breast cancer risk (1, 2). It contributes about 10% of all postmenopausal breast cancer incidents in the United States (3). Obesity disproportionately affects African American (AA) women, where AA women have notably the highest prevalence of obesity and experience the continuing rise (4–6). This trend may reflect increased postmenopausal breast cancer incidence observed among AA women, whereas it has been stable for White women (2, 7–9). During 1999 through 2013, breast cancer incidence among women aged 50 to 59 years decreased slower among AA women (−0.1% per year) compared with White women (−1.7% per year). Furthermore, rates of breast cancer incidence among individuals aged 60 to 79 years increased for AA women, whereas the rates decreased for White women (8). The continuing trend of increased obesity in AA than White women explain the existing difference in breast cancer incidence and may results in widening the racial gap. Notwithstanding the strong epidemiologic evidence that differs considerably by race, biological mechanisms underlying the racial differences in the obesity and postmenopausal breast cancer is yet to be fully elucidated.

Adiponectin is a protein hormone that is secreted by adipose tissue playing a key role in regulating the metabolism of glucose and lipid, adipocyte inflammation, and cell proliferation (10, 11). Adiponectin levels are inversely associated with obesity (12, 13). In obesity, adiponectin resistance is increased with reduced expression of adiponectin receptors (*ADIPOR1* and *ADIPOR2*) in breast cancer cells (14, 15). Consequently, hypoadiponectinemia may predispose to breast cancer development by inhibiting cell apoptosis and enhancing cell proliferation through blocking several downstream signaling pathways, including AMP-activated protein kinase (AMPK) and mitogen-activated protein kinase (MAPK) signaling pathways (15, 16). Observational studies showed that adiponectin levels were lower in women with postmenopausal breast cancer compared to healthy women (17–19). Moreover, lower concentrations of adiponectin were found in AA postmenopausal women compared with White postmenopausal women (20). As such, adiponectin is emerging as a crucial adipokine in breast cancer development in women with obesity, and potentially explains the difference in the breast cancer incidence between AA and White women.

Only a few studies have identified genetic variants (i.e., single nucleotide polymorphisms [SNPs]) that were associated with functional and structural regulation of adiponectin and their association with breast cancer risk; but the findings were inconsistent and conducted mostly in population with European or Asian ancestry (21–25). In particular, rs1501299 in the adiponectin gene (*ADIPOQ*) was associated with an increased risk of breast cancer in some studies (21, 23), but not in others (22). No consensus could be reached for *ADIPOQ* rs2241766, where a positive, a negative, and no associations with breast cancer risk have been reported across different studies (21–24). Of these studies, one study was conducted in AA women reporting that only *ADIPOQ* rs1501299 was associated with increased breast cancer incidence (23). A pressing need remains to consider SNPs in other genes that were found to be associated with adiponectin levels including *CDH13* (26), *FER* (27), and *ARL15* (28) as the existing studies solely examined SNPs in adiponectin and its receptor genes. Investigating SNPs in the *ADIPOQ*, *ADIPOR1*, *ADIPOR2*, and other genes, and further examining the role of obesity in the association between the adiponectin-related SNPs and breast cancer risk could further shed light on the gene-obesity interrelated molecular pathway of adiponectin in breast cancer development.

The purpose of this study was to examine the effects of candidate SNPs that were previously confirmed by genome-wide association and independent replication studies of serum adiponectin levels on breast cancer risk among AA postmenopausal women, who are vulnerable to both high incidence of obesity and breast cancer risk, using a large prospective cohort study from the Women’s Health Initiative (WHI) (23, 26, 27, 29–35). We hypothesized that the effects of candidate SNPs on breast cancer risk differs by obesity status, and therefore, investigated adiponectin-related SNPs that interact with obesity for their associations with breast cancer risk (**Supplementary Figure 1**).

## Methods

### Study Population

The study included postmenopausal women aged 50 to 79 years enrolled in the WHI Clinical Trial and Observational Study that was conducted from 1993 to 2005. The details of its study design and method are described elsewhere (36, 37). Briefly, the WHI was designed to identify risk factors for major causes of morbidity and mortality and to develop prevention strategies for chronic diseases among postmenopausal women. Women were eligible for the WHI study if they were aged 50 to 79 years at the study enrollment; postmenopausal; and likely to reside in the same area for at least 3 years. Genome-wide genotype data have been collected on a subset of participants after obtaining additional consent for genetic studies. We included postmenopausal women enrolled in the WHI SNP Health Association Resources (SHARe) providing the molecular and genetic data of AA and Hispanic women (38).

For the purpose of our study, subjects must meet the following inclusion criteria to be included in the study analysis: the subjects (i) were AA postmenopausal women aged 50 to 79 years; (ii) without a diagnosis of cancer at the time of study enrollment (except non-melanoma skin cancer); and (iii) reported at least one of four physical measurements (i.e., height, weight, waist, and hip). Assuming that those who ended participation early are more likely to have incomplete outcome information leading to potential follow-up bias, the study excluded those who had been followed up for less than 1 year. In addition, individuals who had developed invasive breast cancer within the 1-year follow-up period were excluded to avoid the potential effects of reverse causation between obesity and invasive breast cancer risk.

**Figure 1:**
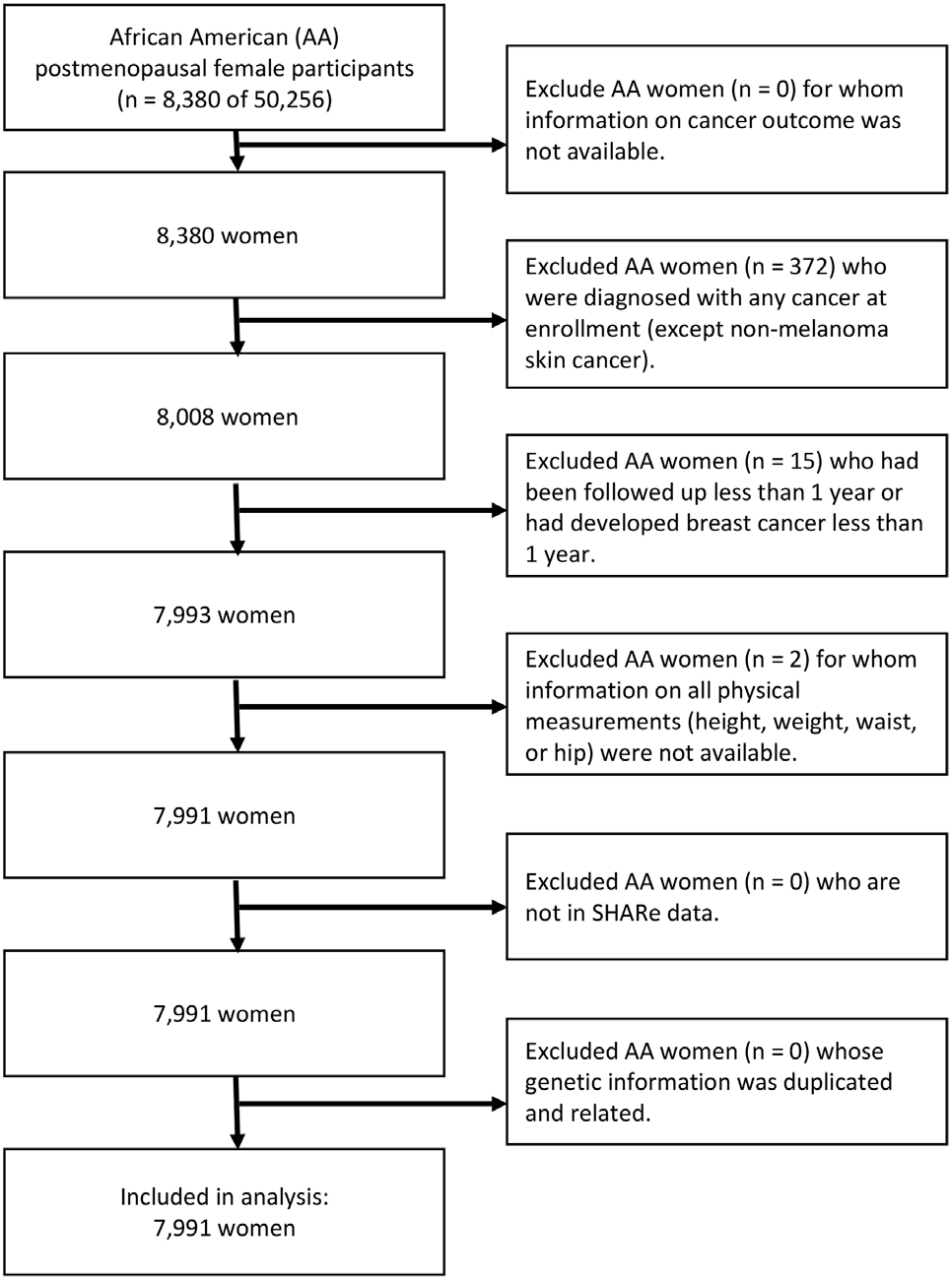
Flow diagram of analytic cohort.

Of 50,256 participants, a total of 8,380 identified their race or ethnicity as AA. We excluded 372 subjects who reported a diagnosis of any type of cancer at the time of enrollment, and two subjects who were missing information on all four physical measurements. Further, we excluded 15 participants who had developed invasive breast cancer within the 1-year follow-up period. There was no withdrawal or cessation of participation within the 1-year follow-up period. After applying the eligibility criteria, a total of 7,991 subjects were included in the analysis. Of 7,991 participants, 402 (5.0%) of the eligible women, greater than the breast cancer incidence of AA postmenopausal women in the US (9), developed invasive breast cancer (**Figure 1**).

### Breast Cancer Outcome

Self-reported invasive breast cancer cases were verified by adjudication of medical records in all participants of all phases of the WHI studies (39). As a result of the comprehensive outcome-assessment procedure, we did not have missing outcomes. Given that each type of breast cancer has distinct etiologies and prognoses for patients, the current study only included participants with primary invasive breast cancer. The participants were followed up from the date of enrollment to invasive breast cancer diagnosis, death, or end of follow-up.

### Obesity Status: Body Mass Index, Waist-to-Hip Ratio, and Waist Circumference

We used three indices measuring body fat based on anthropometric measurements: BMI, waist-to-hip ratio (WHR), and waist circumference (WC). Each index was considered as a potential effect modifier to estimate its effect on the association between adiponectin-related SNPs and breast cancer among AA women. Also, these indices were each separately considered as a confounder of the relationship. Trained research personnel measured anthropometric measurements as continuous variables at the baseline (40). We used internationally recommended cutoff points for assessing adiposity-related risk (41, 42). BMI was categorized to define overall obesity with the following scale: underweight (< 18.5 kg/m^2^), normal (18.5–24.9 kg/m^2^), overweight (25–29.9 kg/m^2^), and obesity (≥ 30 kg/m^2^) (41). WHR used a cutoff of 0.85 (42), and WC used a cutoff of 88 cm in women to define abdominal obesity (41).

### Adiponectin-Related SNPs

We conducted a candidate SNP approach, focusing on variants previously identified in GWA and replication studies of serum adiponectin levels (23, 26, 27, 29–35). Using the annotation file from the Affymetrix Genome-Wide Human SNP Array 6.0, we identified a total of 32 candidate SNPs (23, 26, 27, 29–35) and extracted them from the WHI SHARe dataset using the PLINK 1.9 software (**Supplementary Table 1**). Quality control was performed to exclude SNPs with a call rate less than 90%, a minor allele frequency less than 1%, and a Hardy-Weinberg equilibrium among AA women using a p-value cutoff of (38). We identified 3 SNPs in *ADIPOQ* (rs3774261, rs6444174, and rs6773957) that were in high linkage disequilibrium at a pairwise r^2^ threshold of 0.80. Of the 32 candidate SNPs, 8 SNPs were located within or nearby *ADIPOQ* or adiponectin receptor genes (23, 29). The other 24 SNPs, which may support the function of transcriptional control structures or indirectly regulate adiponectin expression, were found within non-adiponectin–specific or uncharacterized genes (27, 29, 32, 33, 35, 43).

### Statistical Analysis

Baseline characteristics were compared across breast cancer status using a chi-square test for categorical variables and a t-test for continuous variables. We estimated hazard ratio (HR) and its 95% confidence intervals (CIs) for an effect of each SNP in predicting breast cancer development using a Cox proportional hazards regression model. Prior to fitting the model, the proportional hazard assumption was verified using the Schoenfeld residuals.

For each SNP, two sets of adjusted models were used, with the first adjusting for only age at baseline (Model 1) and the second adjusting for all covariates (Model 2). Covariates included in the analysis as potential confounding factors were measured at the baseline: age at baseline (year), family income (<$34,999, $35,000–$100,000, and ≥$100,000), employment status (yes *vs.* no), depressive symptom (depression scale ranging from 0 to 1 with a higher score indicating greater depressive severity), smoking status (ever smoke *vs.* no), age at menopause (year), number of pregnancies (never pregnant, 1 pregnancy, 2–4 pregnancies, and ≥5 pregnancies), exogenous estrogen use ever (yes *vs.* no), exogenous estrogen and progesterone use ever (yes *vs.* no), diabetic status (yes *vs.* no), dietary alcohol per day (gram), dietary total fat (gram), and physical activity (metabolic equivalent of task [MET] hours per week). We performed a complete case analysis excluding study participants with missing data in covariates. All 7,991 participants had data on age at baseline for fitting model 1, and a total of 6,121 participants (77%) were eligible for fitting model 2.

We compared crude and adjusted HRs to assess the effect of obesity as a confounding factor on the association between adiponectin-related SNPs and breast cancer risk. BMI, WHR, and WC are highly correlated, and thus, each index was entered individually in the regression models. A change greater than or equal to 10% indicates the presence of a confounding effect (44). For interaction analysis, two strategies were employed to assess the role of obesity on the relationship between adiponectin-related SNPs and breast cancer risk: (i) stratified analysis and (ii) analysis of the joint effects. The stratified analysis evaluates effect modification by comparing strata-specific HRs to one another and to the crude estimates. The analyses were performed separately for each index of obesity. Next, we calculated the relative excess risk due to interaction (RERI) to assess the joint effects of obesity and adiponectin-related SNPs on breast cancer risk on the additive scale with its 95% CIs obtained by the delta method (45). RERI equals 0 in the absence of additive interaction. Any departure from 0 indicates the presence of additive interaction. All statistical tests considered two-tailed *p* values less than 0.05 to be indicative of statistical significance. To account for the correction of multiple comparisons, we additionally applied the Benjamini and Hochberg procedure and controlled the false discovery rate at q-value of 0.05 in each adiponectin-related SNP (46). The R3.6.0 (dplyr, survival, epiR, and msm packages) was used.

## Results

### Baseline Characteristics

Of 7,991 subjects, 402 (5.0%) reported developing breast cancer (**Table 1**). The overall mean age at the baseline was 60.9 years (SD, 6.8 years) with a mean follow-up year of 14.5 years (SD, 3.15 years). The mean BMI was 31.0 kg/m^2^ (SD, 6.3 kg/m^2^), the mean WHR was 0.82 (SD, 0.07), and the mean WC was 91.3 cm (SD, 13.3 cm). Characteristics of participants were generally balanced between those with and without breast cancer.

**Table 1:** Characteristics of participants by invasive breast cancer status.

	Total		Invasive breast cancer		No invasive breast cancer		
	(N = 7,991)		(N = 402)		(N = 7,589)		
	N	%	N	%	N	%	p^a^
Age group at baseline (year)							0.83
≤ 59	3,592	45	179	44.5	3,413	45	
60–69	3,423	42.8	177	44	3,246	42.8	
≥ 70	976	12.2	46	11.4	930	12.3	
BMI classification (kg/m2)							0.53
Underweight (< 18.5)	24	0.3	0	0	24	0.3	
Normal weight (18.5–24.9)	1,240	15.6	65	16.2	1,175	15.6	
Overweight (25–29.9)	2,681	33.8	127	31.7	2,554	33.9	
Obesity (≥ 30)	3,993	50.3	209	52.1	3,784	50.2	
WHR classification							0.27
< 0.85	5,342	67.1	259	64.6	5,083	67.2	
≥ 0.85	2,618	32.9	142	35.4	2,476	32.8	
WC classification (cm)							0.30
< 88	3,440	43.1	163	40.6	3,277	43.3	
≥ 88	4,534	56.9	238	59.4	4,296	56.7	
Family income ($)							0.40
< 34,999	3,767	50.2	178	47.1	3,589	50.4	
35,000–100,000	3,347	44.6	177	46.8	3,170	44.5	
≥ 100,000	390	5.2	23	6.1	367	5.2	
Employment status							0.78
No	4,201	56.1	207	55.3	3,994	56.1	
Yes	3,294	43.9	167	44.7	3,127	43.9	
Smoking status							0.81
No	3,900	49.3	195	48.7	3,705	49.4	
Ever smoke	4,006	50.7	205	51.2	3,801	50.6	
Number of pregnancies							0.71
Never pregnant	589	7.4	34	8.5	555	7.4	
1 pregnancy	795	10	44	11.1	751	10	
2–4 pregnancies	4,199	53	204	51.3	3,995	53.1	
≥ 5 pregnancies	2,339	29.5	116	29.1	2,223	29.5	
Exogenous estrogen use ever							0.17
No	5,336	66.8	281	69.9	5,055	66.6	
Yes	2,654	33.2	121	30.1	2,533	33.4	
Exogenous estrogen + progesterone use ever							0.25
No	7,059	88.3	348	86.6	6,711	88.4	
Yes	931	11.7	54	13.4	877	11.6	
Diabetic status							0.62
No	7,048	88.3	350	87.5	6,698	88.3	
Yes	935	11.7	50	12.5	885	11.7	
** **	**Mean**	**SD**	**Mean**	**SD**	**Mean**	**SD**	**p** ^a^
Age at baseline (year)	60.9	6.8	60.9	6.7	60.9	6.8	0.91
BMI (kg/m^2^)	31	6.3	31.2	6	31	6.3	0.67
WHR	0.82	0.074	0.82	0.073	0.82	0.074	0.33
WC (cm)	91.3	13.3	91.8	12.4	91.2	13.3	0.40
Dietary alcohol per day (g)	2.4	9.01	1.9	5.4	2.4	9.2	0.30
Dietary total fat (g)	64.2	44.7	62.1	37.8	64.3	45	0.35
Depressive symptom^b^	0.047	0.141	0.044	0.127	0.048	0.142	0.60
Physical activity (METs hours per week^c^)	9.84	12.7	9.6	12.5	9.9	12.8	0.68
Age at menopause (year)	46.6	7.3	46.9	7.8	46.6	7.3	0.44

BMI, body mass index; WHR, waist-to-hip ratio; WC, waist circumference; MET, metabolic equivalent of task.

a From a chi-squared test for categorical variables and a t-test for continuous variables.

b Depression scale ranging from 0 to 1 with a higher score indicating a greater depressive severity.

c The intensity of physical activity is represented in a MET unit by measuring the amount of oxygen consumption during exercise.

### The Association Between Adiponectin-Related SNPs and Breast Cancer Risk

Among 32 adiponectin-related SNPs (**Supplementary Table 2**), three candidate SNPs were observed to have potential association between genotype and breast cancer risk (**Table 2**). Without adjusting for obesity, the heterozygous TC genotype of *OR8S1* rs11168618 (effect allele/reference allele: T/C) was correlated with a lower risk of breast cancer compared to the major CC genotype (HR, 0.65; 95% CI, 0.48–0.88) in model 1. The heterozygous TC genotype of *EIF4A2* rs266719 (T/C) decreased breast cancer risk compared with the major CC genotype in model 2 (HR, 0.65; 95% CI, 0.44–0.95). The heterozygous CA genotype of *KCNK9* rs2468677 (C/A) had increased breast cancer risk compared with the major AA genotype; however, it was found to be statistically significant only in model 2 with an additional adjustment for BMI (HR, 1.35; 95% CI, 1.00–1.80). After adjustments for multiple testing, those SNPs did not reach the significance level. Further, the assessment of confounding by BMI, WHR, and WC on the SNP-breast cancer relationship revealed that a confounding effect is unlikely to be a concern.

**Table 2:** Associations of adiponectin-related SNPs and postmenopausal invasive breast cancer risk with or without adjusting for BMI, WHR, and WC.

		No adjustment for obesity status				Additional adjustment for BMI				Additional adjustment for WHR				Additional adjustment for WC			
		Model 1^a^		Model 2^b^		Model 1^a^		Model 2^b^		Model 1^a^		Model 2^b^		Model 1^a^		Model 2^b^	
**Genotype**	**brca/no**	**HR (95% CI)**	P^c^	**HR (95% CI)**	P^c^	**HR (95% CI)**	P^c^	**HR (95% CI)**	P^c^	**HR (95% CI)**	P^c^	**HR (95% CI)**	P^c^	**HR** **(95% CI)**	P^c^	**HR** **(95% CI)**	P^c^
rs266719																	
CC	306/5,671	1		1		1		1		1		1		1		1	
TC	41/1,036	0.74 (0.54–1.03)	0.07	**0.65 (0.44**–**0.95)**	**0.03**	0.74 (0.54–1.03)	0.07	**0.65 (0.44**–**0.95)**	**0.03**	0.74 (0.54–1.03)	0.07	**0.65 (0.44**–**0.96)**	**0.03**	0.74 (0.54, 1.03)	0.07	**0.65 (0.44, 0.95)**	**0.03**
TT	5/54	1.65 (0.68–4.00)	0.27	1.84 (0.68–4.95)	0.23	1.65 (0.68–4.00)	0.27	1.83 (0.68–4.94)	0.23	1.66 (0.69–4.02)	0.26	1.85 (0.69–4.98)	0.22	1.65 (0.68, 4.00)	0.27	1.84 (0.68, 4.95)	0.23
rs2468677																	
AA	81/1,841	1		1		1		1		1		1		1		1	
CA	188/3,353	1.27 (0.98–1.64)	0.08	1.33 (0.99–1.77)	0.06	1.28 (0.99–1.66)	0.07	**1.35 (1.01**–**1.80)**	**<0.05**	1.27 (0.98–1.64)	0.08	1.33 (0.99–1.77)	0.06	1.27 (0.98, 1.64)	0.08	1.33 (1.00, 1.77)	0.05
CC	83/1,569	1.20 (0.88–1.63)	0.25	1.14 (0.80–1.61)	0.47	1.21 (0.89–1.65)	0.22	1.15 (0.81–1.64)	0.43	1.20 (0.89–1.63)	0.24	1.14 (0.81–1.62)	0.45	1.20 (0.88, 1.63)	0.25	1.14 (0.80, 1.61)	0.47
rs11168618																	
CC	297/5,324	1		1		1		1		1		1		1		1	
TC	49/1,338	**0.65 (0.48**–**0.88)**	**0.01**	0.73 (0.53–1.01)	0.05	**0.65 (0.48**–**0.88)**	**0.01**	0.74 (0.53–1.02)	0.06	**0.65 (0.48**–**0.87)**	**<0.01**	**0.72 (0.52**–**1.00)**	**<0.05**	**0.65 (0.48, 0.88)**	**0.01**	0.73 (0.53, 1.01)	0.05
TT	6/102	1.02 (0.46–2.30)	0.96	1.08 (0.45–2.63)	0.86	1.01 (0.45–2.27)	0.98	1.08 (0.45–2.63)	0.86	1.02 (0.46–2.29)	0.96	1.09 (0.45–2.65)	0.85	1.02 (0.46, 2.30)	0.96	1.09 (0.45, 2.66)	0.84

Boldface text indicates statistical significance at P < 0.05.

Chr, chromosome; brca, invasive breast cancer; BMI, body mass index; WHR, waist-to-hip ratio; WC, waist circumference; CI, confidence interval

a Adjusted for age only.

b Adjusted for age, dietary alcohol (g), diabetes, dietary fat (g), depression scale, energy expenditure, employment status, ever smoking status, number of pregnancies, age at menopause, income status, unopposed estrogen use ever, unopposed estrogen + progesterone use ever.

c Results do not reach the significance level (q < 0.05) after adjustments for multiple testing with the Benjamini and Hochberg procedure.

### BMI, WHR, and WC as Effect Modifiers of the Association Between Adiponectin-Related SNPs and Breast Cancer Risk

**Tables 3**–**5** present analysis stratified by BMI (under/normal weight, overweight, and obesity), WHR (<0.85 *vs.* ≥0.85), and WC (<88 cm *vs.* ≥88 cm), respectively. The effects of obesity status on the relationship between several SNPs and breast cancer differed between strata. In model 1, the heterozygous TC genotype in *OR8S1* rs11168618 (T/C) was inversely associated with breast cancer risk among individuals with under/normal weight, overweight, WHR <0.85, and WC <88 cm. However, the significance was no longer observed in model 2. There was also a possible interaction of BMI ≥30 kg/m^2^ with the heterozygous TC genotype in *OR8S1* rs11168618 (T/C) (RERI, 0.58; 95% CI, 0.35–0.80).

**Table 3:** Association between adiponectin-related SNPs and postmenopausal invasive breast cancer risk, by BMI status.

		Under/Normal Weight				Overweight					Obesity				
Genotype		Model 1a		Model 2b		Model 1a		Model 2b		RERI (95% CI)c	Model 1a		Model 2b		RERI (95% CI)c
	brca/no	HR (95% CI)	p	HR (95% CI)	p	HR (95% CI)	p	HR (95% CI)	p		HR (95% CI)	p	HR (95% CI)	p	
rs2791553															
CC	106/2,050	1		1		1		1			1		1		
		0.71	0.24	0.63	0.15	1.53	0.06	1.37	0.20	0.57	1.04	0.83	0.99	0.96	0.37
TC	191/3,329	(0.40–1.26)		(0.33–1.18)		(0.99–2.38)		(0.84–2.23)		(0.31–0.83)	(0.75–1.44)		(0.69, 1.43)		(0.19, 0.55)
		0.55	0.14	0.56	0.20	1.11	0.72	1.24	0.50	0.58	0.68	0.1	0.66	0.1	0.15
TT	55/1,385	(0.24–1.23)		(0.23–1.35)		(0.62–1.99)		(0.67–2.29)		(0.25–0.92)	(0.43–1.07)		(0.40, 1.09)		(-0.26, 0.56)
rs4301033															
GG	236/4,455	1		1		1		1			1		1		
		0.92	0.79	0.94	0.86	0.73	0.15	0.73	0.22	−0.14	1.05	0.78	1.12	0.52	0.17
AG	99/2,049	(0.51–1.65)		(0.49–1.81)		(0.47–1.12)		(0.45–1.20)		(−0.46 to 0.18)	(0.76–1.44)		(0.79, 1.61)		(-0.34, 0.69)
		0.54	0.54	0.64	0.67	1.82	0.11	2.12	<0.05	1.4	1.18	0.66	1.4	0.39	0.74
AA	17/249	(0.074–3.92)		(0.087–4.76)		(0.88–3.75)		(1.01–4.44)		(−0.15 to 2.96)	(0.58–2.41)		(0.65, 3.03)		(0.001, 1.49)
rs266719															
CC	306/5,671	1		1		1		1			1		1		
		1.07	0.84	0.99	0.97	0.67	0.18	1.21	0.05	−0.61	0.66	0.10	1.29	0.10	-0.50
TC	41/1,036	(0.55–2.08)		(0.47–2.06)		(0.38–1.20)		(0.77–1.90)		(−1.15 to −0.069)	(0.40–1.08)		(0.89, 1.81)		(-0.99, -0.01)
		0.00	0.99	0.00	0.99	0.79	0.82	1.34	0.89	1.17	2.71	0.05	0.99	0.10	2.79
TT	5/54	(0.00 to Inf)		(0.00 to Inf)		(0.11–5.68)		(0.73–2.46)		(0.76–1.57)	(1.01–7.32)		(0.58, 1.70)		(2.38, 3.20)
rs3821799															
TT	112/2,140	1		1		1		1			1		1		
		0.55	0.04	0.42	0.01	1.44	0.10	1.49	0.11	0.77	0.93	0.66	0.86	0.43	0.45
CT	169/3,330	(0.30–0.98)		(0.22–0.81)		(0.93–2.23)		(0.92–2.43)		(0.49–1.06)	(0.66–1.30)		(0.59, 1.25)		(0.13, 0.77)
		0.69	0.33	0.59	0.21	1.38	0.24	1.41	0.25	0.57	1.02	0.92	1.04	0.88	0.37
CC	71/1,294	(0.33–1.46)		(0.26–1.34)		(0.81–2.37)		(0.78–2.57)		(0.18–0.95)	(0.68–1.54)		(0.66, 1.64)		(0.08, 0.65)
rs3774261d															
TT	110/2,123	1		1		1		1			1		1		
		0.59	0.07	0.40	0.01	1.24	0.32	1.24	0.38	0.67	1.02	0.89	0.99	0.95	0.58
CT	166/3,263	(0.33–1.05)		(0.21–0.77)		(0.81–1.91)		(0.77–2.01)		(0.39–0.99)	(0.73–1.44)		(0.68, 1.45)		(0.28, 0.88)
		0.66	0.28	0.63	0.24	1.28	0.35	1.31	0.35	0.5	1.12	0.58	1.1	0.70	0.4
CC	76/1,372	(0.31–1.39)		(0.29–1.37)		(0.76–2.14)		(0.74–2.32)		(0.14–0.86)	(0.75–1.69)		(0.69, 1.74)		(0.11, 0.68)
rs6444174d															
TT	249/4,940	1		1		1		1			1		1		
		1.39	0.26	1.82	0.06	0.7	0.13	0.76	0.28	−1	1.26	0.17	1.22	0.29	-0.52
CT	90/1,658	(0.78–2.46)		(0.98–3.35)		(0.43–1.11)		(0.46–1.25)		(−2.27 to 0.27)	(0.91–1.76)		(0.84, 1.76)		(-2.78, 1.74)
		0.00	1.00	0.00	1.00	1.53	0.36	1.19	0.77	1.22	2.04	0.05	2.26	0.04	2.42
CC	13/164	(0.00 to Inf)		(0.00 to Inf)		(0.62–3.76)		(0.37–3.82)		(0.72–1.72)	(0.99–4.18)		(1.04, 4.88)		(1.91, 2.93)
rs6773957d															
TT	104/2,034	1		1		1		1			1		1		
		0.55	0.05	0.39	0.01	1.3	0.24	1.32	0.27	0.74	1.05	0.78	1.04	0.85	0.62
CT	171/3,349	(0.30–0.99)		(0.20–0.75)		(0.84–2.01)		(0.81–2.16)		(0.43–1.04)	(0.74–1.48)		(0.71, 1.53)		(0.32, 0.92)
		0.71	0.35	0.6	0.20	1.32	0.30	1.37	0.28	0.54	1.13	0.57	1.12	0.63	0.44
CC	77/1,379	(0.34–1.47)		(0.27–1.32)		(0.78–2.23)		(0.77–2.45)		(0.17–0.92)	(0.75–1.71)		(0.70, 1.79)		(0.14, 0.74)
rs13434995															
AA	253/5,032	1		1		1		1			1		1		
		1.69	0.07	2.2	0.01	1.13	0.56	1.14	0.59	−1.04	0.97	0.85	0.99	0.98	-1.29
GA	91/1,617	(0.96–2.97)		(1.19–4.07)		(0.75–1.72)		(0.71–1.82)		(−4.89 to 2.81)	(0.68–1.37)		(0.68, 1.46)		(-6.42, 3.83)
		3.94	0.02	7.14	<0.01	2.37	0.09	2.73	0.06	−3.66	0.3	0.23	0.41	0.38	-6.08
GG	8/115	(1.21–12.86)		(2.05–24.86)		(0.87–6.49)		(0.96–7.75)		(−48.02 to 40.71)	(0.042–2.16)		(0.057, 2.96)		(-18.10, 5.95)
rs10012953															
TT	238/4,540	1		1		1		1			1		1		
		0.75	0.36	0.91	0.77	1.03	0.87	1.06	0.80	0.19	0.99	0.95	1.13	0.51	0.22
CT	100/1,987	(0.40–1.39)		(0.47–1.77)		(0.70–1.54)		(0.68–1.64)		(−0.16, 0.53)	(0.71–1.37)		(0.79, 1.62)		(-0.18, 0.63)
		0.44	0.41	0.57	0.59	0.7	0.55	0.87	0.81	0.31	1.63	0.16	2.14	0.03	1.57
CC	13/230	(0.06–3.16)		(0.078–4.25)		(0.22–2.23)		(0.27–2.78)		(−0.30, 0.91)	(0.83–3.22)		(1.07, 4.25)		(0.30, 2.84)
rs10447248															
CC	245/4,881	1		1		1		1			1		1		
		1.46	0.20	1.6	0.14	1.01	0.98	1.12	0.62	−0.37	1.01	0.96	1.05	0.82	-0.49
TC	94/1,728	(0.82–2.57)		(0.85–3.01)		(0.66–1.52)		(0.71–1.75)		(−1.92 to 1.18)	(0.72–1.42)		(0.71, 1.53)		(-2.18, 1.20)
		1.7	0.47	2.2	0.30	0.98	0.97	0.41	0.37	−1.59	2.20	0.03	2.53	0.02	0.54
TT	13/152	(0.41–7.05)		(0.50–9.67)		(0.31–3.11)		(0.056–2.93)		(−3.89 to 0.72)	(1.08–4.49)		(1.17, 5.45)		(-10.66, 11.73)
rs998584															
CC	205/4,106	1		1		1		1			1		1		
		0.87	0.64	1	1.00	1.03	0.90	1.03	0.89	0.064	1.23	0.19	1.36	0.08	0.34
AC	129/2,313	(0.49–1.55)		(0.53–1.88)		(0.69–1.52)		(0.67–1.60)		(−0.23 to 0.36)	(0.91–1.66)		(0.97, 1.90)		(-0.38, 1.06)
		0.95	0.94	0.79	0.75	1.94	0.04	2.45	0.01	1.65	0.44	0.11	0.58	0.29	-0.28
AA	18/344	(0.29–3.10)		(0.18–3.36)		(1.02–3.67)		(1.27–4.72)		(0.32–2.98)	(0.16–1.20)		(0.21, 1.58)		(-1.04, 0.47)
rs11168618															
CC	297/5,324	1		1		1		1			1		1		
		0.37	0.03	1	0.05	0.52	0.02	0.67	0.20	0.35	0.87	0.49	1.01	0.77	0.58
TC	49/1,338	(0.15–0.93)		(0.54–1.86)		(0.30–0.91)		(0.42–1.05)		(0.03–0.68)	(0.59–1.29)		(0.71, 1.44)		(0.35, 0.80)
		0.76	0.79	0.73	0.83	0.97	0.97	1.22	0.70	0.38	1.1	0.87	0.89	0.95	-0.022
TT	6/102	(0.11–5.55)		(0.27–1.96)		(0.24–3.93)		(0.70–2.13)		(−0.90 to 1.65)	(0.35–3.45)		(0.53, 1.50)		(-1.24, 1.19)

Boldface text indicates statistical significance at P < 0.05.

Chr, chromosome; brca, invasive breast cancer; BMI, body mass index; WHR, waist-to-hip ratio; WC, waist circumference; CI, confidence interval; NA, not applicable; RERI, relative excess risks due to interaction.

a Adjusted for age.

b Adjusted for age, dietary alcohol (g), diabetes, dietary fat (g), depression scale, energy expenditure, employment status, ever smoking status, number of pregnancies, age at menopause, income status, unopposed estrogen use ever,unopposed estrogen + progesterone use ever.

c RERI and its 95% Cis were calculated for fully adjusted Cox models (aHR2) only

d High linkage disequilibrium (r2 > 0.80) was found between all pairs of these three SNPs in ADIPOQ.

**Table 4:** Association between adiponectin-related SNPs and postmenopausal invasive breast cancer risk, by WHR status.

		WHR < 0.85				WHR ≥ 0.85				
Genotype		Model 1^a^		Model 2^b^		Model 1^a^		Model 2^b^		RERI (95% CI)^c^
	brca/no	HR (95% CI)	p	HR (95% CI)	p	HR (95% CI)	p	HR (95% CI)	p	
rs4301033										
GG	236/4,455	1		1		1		1		
		1.02	0.90	1.03	0.85	0.75	0.18	0.79	0.31	−0.31
AG	99/2,049	(0.77–1.36)		(0.75, 1.43)		(0.49–1.14)		(0.50–1.25)		(−1.08 to 0.45)
		1.55	0.13	**1.9**	**0.04**	0.84	0.74	0.99	0.99	−0.86
AA	17/249	(0.88–2.73)		**(1.04–3.46)**		(0.31–2.30)		(0.36–2.72)		(−4.58 to 2.86)
rs10517133										
GG	293/5,679	1		1		1		1		
		0.8	0.26	0.86	0.49	**1.57**	**0.04**	1.37	0.19	0.55
CG	56/1,027	(0.54–1.18)		(0.56–1.32)		**(1.04–2.39)**		(0.86–2.20)		(−0.26 to 1.35)
		1.32	0.63	1.06	0.93	0.00	0.99	0.00	0.99	NA
CC	3/52	(0.42–4.14)		(0.26–4.30)		(0.00 to Inf)		(0.00 to Inf)	
rs13434995										
AA	253/5,032	1		1		1		1		
		1.15	0.34	1.29	0.13	1.05	0.81	1.06	0.81	−0.2
GA	91/1,617	(0.86–1.55)		(0.93–1.78)		(0.69–1.60)		(0.67–1.67)		(−1.60 to 1.20)
		1.92	0.09	**2.76**	**0.01**	0.44	0.42	0.52	0.52	−2.56
GG	8/115	(0.90–4.10)		**(1.23**–**5.96)**		(0.062–3.18)		(0.072–3.76)		(−7.62 to 2.51)
rs10447248										
CC	245/4,881	1		1		1		1		
		**1.44**	**0.01**	**1.65**	**<0.01**	**0.51**	**0.01**	**0.46**	**0.01**	−**1.54**
TC	94/1,728	**(1.09**–**1.90)**		**(1.21**–**2.24)**		**(0.31**–**0.86)**		**(0.26**–**0.82)**		**(**−**2.80 to** −**0.28)**
		1.67	0.16	1.74	0.19	1.59	0.31	1.42	0.50	−0.062
TT	13/152	(0.82–3.41)		(0.76–3.97)		(0.65–3.90)		(0.52–3.89)		(−8.77 to 8.65)
rs11168618										
CC	297/5,324	1		1		1		1		
		**0.67**	**0.03**	0.79	0.24	0.61	0.06	0.59	0.07	−0.28
TC	49/1,338	**(0.46**–**0.97)**		(0.53–1.17)		(0.36–1.02)		(0.34–1.04)		(−0.70 to 0.14)
		1.03	0.95	1.03	0.97	1.02	0.98	1.16	0.84	0.24
TT	6/102	(0.38–2.78)		(0.33–3.23)		(0.25–4.11)		(0.28–4.76)		(−2.91 to 3.39)
rs10847980										
TT	192/3,629	1		1		1		1		
		1.07	0.65	1.03	0.85	0.78	0.19	0.69	0.08	−0.52
GT	130/2,635	(0.81–1.41)		(0.75–1.42)		(0.53–1.13)		(0.46–1.05)		(−1.36 to 0.32)
		**1.7**	**0.02**	**1.73**	**0.02**	0.37	0.05	**0.31**	**0.049**	−**1.7**
GG	30/498	**(1.11**–**2.60)**		**(1.07**–**2.78)**		(0.14–1.01)		**(0.098**–**0.99)**		**(**−**3.13 to** −**0.26)**
rs3865188										
TT	123/2,644	1		1		1		1		
		1.12	0.44	1.1	0.57	1.38	0.12	1.42	0.12	0.3
AT	175/3,149	(0.84–1.48)		(0.80–1.51)		(0.92–2.05)		(0.92–2.20)		(−0.43 to 1.03)
		1.03	0.88	0.94	0.80	1.58	0.09	**1.81**	**0.04**	0.81
AA	54/971	(0.69–1.54)		(0.59–1.50)		(0.93–2.69)		**(1.02**–**3.19)**		(−0.32 to 1.93)

Boldface text indicates statistical significance at P < 0.05.

Chr, chromosome; brca, invasive breast cancer; BMI, body mass index; WHR, waist-to-hip ratio; WC, waist circumference; CI, confidence interval; NA, not applicable; RERI, relative excess risks due to interaction.

a Adjusted for age.

b Adjusted for age, dietary alcohol (g), diabetes, dietary fat (g), depression scale, energy expenditure, employment status, ever smoking status, number of pregnancies, age at menopause, income status, unopposed estrogen use ever, unopposed estrogen + progesterone use ever.

c RERI and its 95% Cis were calculated for fully adjusted Cox models (aHR2) only.

**Table 5:** Association between adiponectin-related SNPs and postmenopausal invasive breast cancer risk, by WC status.

		WHR < 0.85				WHR ≥ 0.85				
Genotype		Model 1^a^		Model 2^b^		Model 1^a^		Model 2^b^		RERI (95% CI)^c^
	brca/no	HR (95% CI)	p	HR (95% CI)	p	HR (95% CI)	p	HR (95% CI)	p	
rs266719										
CC	306/5,671	1		1		1		1		
		0.79	0.33	0.7	0.20	0.7	0.12	0.59	0.06	-0.14
TC	41/1,036	(0.49–1.27)		(0.41–1.21)		(0.45–1.10)		(0.34–1.03)		(-0.44, 0.16)
		0.00	0.99	0.00	0.99	**2.91**	**0.02**	**3.33**	**0.02**	**3.62**
TT	5/54	(0.00 to Inf)		(0.00 to Inf)		**(1.20–7.07)**		**(1.23–9.04)**		**(3.34, 3.90)**
rs3774261^d^										
TT	110/2,123	1		1		1		1		
		0.79	0.21	**0.64**	**0.03**	1.16	0.36	1.12	0.54	**0.43**
CT	166/3,263	(0.55–1.14)		**(0.43–**0**.97)**		(0.84–1.60)		(0.78–1.60)		**(0.28, 0.58)**
		0.99	0.99	0.89	0.62	1.14	0.52	1.15	0.53	0.19
CC	76/1,372	(0.64–1.54)		(0.55–1.43)		(0.77–1.69)		(0.74–1.78)		(-0.079, 0.47)
rs6773957^d^										
TT	104/2,034	1		1		1		1		
		0.78	0.19	**0.65**	**0.04**	1.21	0.26	1.19	0.34	**0.47**
CT	171/3,349	(0.54–1.13)		**(0.43–0.98)**		(0.87–1.67)		(0.83–1.71)		**(0.33, 0.62)**
		1.02	0.94	0.88	0.61	1.16	0.46	1.2	0.42	0.23
CC	77/1,379	(0.66–1.57)		(0.55–1.43)		(0.78–1.73)		(0.77–1.87)		(-0.034, 0.50)
rs13434995										
AA	253/5,032	1		1		1		1		
		1.26	0.22	1.31	0.20	1.01	0.91	1.09	0.63	-0.2
GA	91/1,617	(0.88–1.80)		(0.87–1.96)		(0.74–1.41)		(0.77–1.55)		(-1.39, 0.99)
		**2.62**	**0.02**	**3.65**	**<0.01**	0.55	0.41	0.7	0.61	-3.14
GG	8/115	**(1.15–5.98)**		**(1.57–8.47)**		(0.14–2.24)		(0.17–2.82)		(-8.86, 2.57)
rs13358260										
AA	338/6,525	1		1		1		1		
		1.88	**0.06**	**2.05**	**0.04**	0.59	0.29	0.5	0.24	**-1.6**
GA	14/233	(0.99–3.57)		**(1.03–4.06)**		(0.22–1.58)		(0.16–1.58)		**(-3.18, -0.008)**
		0.00	0.99	0.00	0.99	0.00	0.99	NA	–	NA
GG	0/5	(0.00 to Inf)		(0.00 to Inf)		(0.00 to Inf)				
rs592423										
AA	124/2,310	1		1		1		1		
		1.41	0.07	**1.6**	**0.03**	0.77	0.09	0.79	0.17	-0.97
CA	176/3,355	(0.97–2.04)		**(1.05–2.43)**		(0.57–1.04)		(0.57–1.11)		(-3.65, 1.72)
		1.01	0.98	0.84	0.59	0.84	0.39	0.91	0.68	0.004
CC	52/1,099	(0.59–1.72)		(0.44–1.60)		(0.56–1.26)		(0.58–1.42)		(-1.51, 1.52)
rs11168618										
CC	297/5,324	1		1		1		1		
		**0.53**	**0.01**	0.66	0.11	0.75	0.14	0.78	0.26	0.11
TC	49/1,338	**(0.33–0.87)**		(0.40–1.09)		(0.51–1.10)		(0.52–1.20)		(-0.14, 0.35)
		0.74	0.67	0.98	0.98	1.25	0.66	1.19	0.77	0.18
TT	6/102	(0.18–2.99)		(0.24–4.01)		(0.47–3.38)		(0.38–3.74)		(-1.47, 1.82)

Boldface text indicates statistical significance at P < 0.05.

Chr, chromosome; brca, invasive breast cancer; BMI, body mass index; WHR, waist-to-hip ratio; WC, waist circumference; CI, confidence interval; NA, not applicable; RERI, relative excess risks due to interaction.

a Adjusted for age.

b Adjusted for age, dietary alcohol (g), diabetes, dietary fat (g), depression scale, energy expenditure, employment status, ever smoking status, number of pregnancies, age at menopause, income status, unopposed estrogen use ever, unopposed estrogen + progesterone use ever.

c RERI and its 95% Cis were calculated for fully adjusted Cox models (aHR2) only.

d High linkage disequilibrium (r2 > 0.80) was found between these two SNPs in ADIPOQ.

In relation to the *ADIPOQ* gene, the heterozygous CT genotype in rs6773957 (C/T) was negatively associated with breast cancer risk among individuals with under/normal weight by roughly 60% in model 2. An interaction between BMI ≥30 kg/m^2^ and the heterozygous CT genotype was observed with a RERI of 0.62 with 95% CI of 0.32 to 0.92. Among those with WC <88 cm, the heterozygous CT genotype in rs6773957 (C/T) appeared to have a lower risk of breast cancer compared with the major CC genotype (HR, 0.65; 95% CI, 0.43–0.98). WC ≥88 cm showed an RERI of 0.47 with 95% CI of 0.33 to 0.62, suggesting super-additivity for the interaction between *ADIPOQ* rs6773957 (C/T) and WC. In addition, whereas effect alleles in *ADIPOR1* rs2232853 (T/C) were associated with an increased risk of breast cancer among White women (21), its association with breast cancer risk was not found among AA women.

For *FER* rs10447248 (T/C), women with BMI ≥30 kg/m^2^ and the minor TT genotype had increased breast cancer risk in comparison to those with major CC genotype by approximately 2-fold in both model 1 (HR, 2.20; 95% CI, 1.08–4.49) and model 2 (HR, 2.53; 95% CI, 1.17–5.45). When we stratified the analysis by WHR status, different patterns were observed for the association between *FER* rs10447248 (T/C) and breast cancer risk. Carriers of the heterozygous TC genotype had an elevated risk of breast cancer among WHR <0.85 compared with those with the major CC genotype (HR, 1.44; 95% CI, 1.09–1.90), whereas the reduced risk among WHR >0.85 (HR, 0.51; 95% CI, 0.31–0.86) in model 1.

## Discussion

Low circulating levels of adiponectin have been observed in obese individuals and women with postmenopausal breast cancer (12, 13, 17–19). Yet, the genetic mechanisms underlying the association between adiponectin and obesity in breast cancer risk have not been fully elucidated. Our study evaluated the association between genetic variants involved in regulating adiponectin circulating levels and breast cancer risk by obesity status among postmenopausal AA women. We found that heterozygotes of *OR8S1* rs11168618 (T/C) and *EIF4A2* rs266719 (T/C) were negatively associated with breast cancer risk, whereas the heterozygote of *KCNK9* rs2468677 (C/A) had an elevated risk. The crude estimates of breast cancer risk did not differ from the adjusted estimates, thus confounding by obesity is unlikely. The findings suggest that low circulating levels of adiponectin may serve as a risk factor for breast cancer, independent of obesity. Indeed, *in vivo* and *in vitro* studies have demonstrated a direct effect of adiponectin on breast cancer development with and without obesity environment (47). Increased levels of adiponectin attenuated cell proliferation in several breast cancer cell lines, including MCF-7 (48, 49), T47D (48, 50–52), SKBR3 (48), MDA-MB-231 (50, 51), and MCF-10A (53). Furthermore, transgenic mice with adiponectin injection reduced mammary tumorigenesis (50), whereas mice with reduced adiponectin expression led to earlier tumor onset and accelerated tumor growth compared to those with normal expression (54).

The evidence on the association of SNPs in *ADIPOQ* and *ADIPOR1* with breast cancer risk has been inconsistent (21–25). A previous study found that *ADIPOQ* rs17366568 influenced adiponectin plasma levels in non-Hispanic White women but not in AA women (55). In addition, women who carried effect alleles in *ADIPOR1* rs2232853 (T/C) were associated with increased risk of breast cancer in a case-control study that consists of predominantly White women aged 20 to 87 years (21). However, we did not find a significant correlation between this SNP and breast cancer risk among AA women aged 50 to 79 years. These results in part explain the existing racial variations between AA and White women in breast cancer incidence and adiponectin levels (2, 7–9, 20). The findings also support that different adiponectin-related genetic factors may contribute to the increased risk of breast cancer by race. Understanding racial differences in adiponectin-related SNPs by accounting for their associations with adiponectin levels and breast cancer risk is an important area for future research.

We also observed that SNPs in non-adiponectin-specific genes were associated with breast cancer risk, and these associations were modified by obesity. Individuals with the minor TT genotype of *FER* rs10447248 (T/C) and having BMI ≥30 kg/m^2^ had an elevated risk of postmenopausal breast cancer. FER tyrosine kinase increases NF-*κβ* activation and signals interleukin-6 (IL-6) to regulate STAT3 phosphorylation (56, 57), which may explain its relationship with breast cancer risk through adiponectin and obesity. A decline in adiponectin secretion leads to overexpression of pro-inflammatory cytokines, including IL-6 and TNF-α, in an obese individual as a consequence of excess inflammatory response (58). The induction of TNF-α activates NF-*κβ*, which promotes breast cancer development (59, 60). IL-6 activates the Janus kinase-signal transducer and activator of transcription signaling pathway inducing the STAT3 dimer (58, 61, 62). This STAT3 dimer stimulates the transcription of genes strongly associated with the promotion of tumor growth and immunosuppression. This suggests that *FER* rs10447248 may predispose breast cancer by inducing NF-*κβ* and IL-6 to trigger downstream signaling pathways.

*OR8S1* is an olfactory receptor (OR) that belongs to G protein-coupled receptors influencing tumorigenesis (63). An OR is most abundant in not only olfactory sensory neurons in an olfactory epithelium but is also found in tissues throughout the body. In the current study, the effect of *OR8S1* rs11168618 (T/C), which decreases adiponectin levels (33), was inversely associated with breast cancer risk. It has been reported that an activated OR 544 (*Olfr544*) increased adiponectin secretion in 3T3-L1 mouse adipocytes (64). In relation to breast cancer, *OR2B6* and *OR2W3* were ectopically expressed in breast cancer cell lines and breast cancer tissues making them potential biomarkers (65, 66). An activation of ORs in cancer cells promotes apoptosis and inhibits cell proliferation by inducing AMPK or MAPK signaling pathways (65, 67). Given the limited existing evidence on the role of ORs with adiponectin or different types of cancer, we can only speculate that activation of ORs in adipose tissue has an indirect effect on lowering breast cancer risk through increasing adiponectin levels. To our knowledge, only the present study has evaluated *OR8S1* rs11168618 and breast cancer risk.

It is important to note the potential limitations that the study has. Although breast cancer outcome and anthropometric measurements were prospectively measured with strict ascertainment procedure, other variables included in the regression models were mainly obtained from the self-reported questionnaire at the time of enrollment, leading to recall bias. However, there was no difference in distributions of baseline characteristics among cohorts. Missing data were also unavoidable in this study. In particular, information on the family history of breast cancer had a low response rate of 37.4% reducing statistical efficiency of the estimates. Thus, we decided not to include family history of breast cancer to obtain sufficient power while sustaining a potential confounding effect. Low circulating adiponectin levels may contribute to a more aggressive phenotype of breast cancer, ER-negative breast cancer risk compared to ER-positive breast cancer risk (68). Also, reduced breast cancer risk was observed in women with increased high-molecular weight adiponectin levels and lower BMI (14). Nevertheless, we could not further analyze the data by molecular subtypes of breast cancer or by adiponectin isomers due to the small sample sizes. Lastly, the study was limited to postmenopausal AA women harming the generalizability of our results. Despite these drawbacks, we used one of the most extensive data on postmenopausal AA women and conducted the genetic association study of adiponectin concerning postmenopausal breast cancer risk. In many cases, genetic data of a minority racial or ethnic group are not readily available nor have a sufficient sample size to obtain a comfortable statistical efficiency of the estimates. In addition to finding the associations between candidate SNPs in adiponectin genes and breast cancer risk, the study considered other loci in non–adiponectin-specific genes Associated with regulating adiponectin expression. In doing so, we were able to identify genetic variants of circulating adiponectin levels that were not directly considered in previous studies but may predispose to breast cancer development.

In summary, our study evaluated the association between previously identified adiponectin-related SNPs and primary invasive breast cancer risk among AA postmenopausal women. We detected that several adiponectin-related SNPs interacted with obesity, altering the risk of postmenopausal breast cancer. As obese women have an approximately 30% increased risk in developing breast cancer compared with those with normal weight (69), weight management is recommended as breast cancer prevention strategies (70). In light of the evidence, such an intervention would be particularly beneficial to AA postmenopausal women who carry the risk alleles of the adiponectin-related SNPs. Also, the identified SNPs could be used as clinical and genetic predictors of breast cancer in conjunction with obesity for AA postmenopausal women. Future studies are warranted to incorporate genetic variants of other cytokines from adipocytes (e.g., leptin) to unravel the complexity of the underlying mechanisms between obesity and breast cancer risk among AA women. Also, comparing the effects of adiponectin-related SNPs across different racial/ethnic groups can contribute to better understanding of the racial disparity in breast cancer risk. Nonetheless, our findings may assist in reducing the persistent racial gap in breast cancer incidence between AA and White women by examining the role of obesity and adiponectin in postmenopausal breast cancer etiology that may differ by these racial groups.

## Data Availability Statement

The data that support the findings of this study are available in accordance with policies developed by the NHLBI and WHI in order to protect sensitive participant information and approved by the Fred Hutchinson Cancer Research Center, which currently serves as the IRB of record for the WHI. Data requests may be made by emailing helpdesk@WHI.org.

## Ethics Statement

The studies involving human participants were reviewed and approved by the institutional review boards of each participating clinical center of the WHI and the University of California, Los Angeles. The patients/participants provided their written informed consent to participate in this study.

## Author Contributions

GN, Z-FZ, and SJ designed the study. GN and SJ performed the genomic data QC. GN performed the statistical analysis. Z-FZ, JR, HZ, and SJ participated in the study coordination and interpreted the data. SJ supervised the genomic data QC and data analysis and interpretation. SJ secured funding for this project. All authors contributed to the article and approved the submitted version.

## Funding

This study was supported by the National Institute of Nursing Research of the National Institutes of Health under Award Number K01NR017852. GN is supported by the T32 Training Grant in Cancer Epidemiology (T32CA009142) at University of California, Los Angeles. Part of the data for this project was provided by the WHI program, which is funded by the National Heart, Lung, and Blood Institute, the National Institutes of Health, and the U.S. Department of Health and Human Services through contracts HHSN268201100046C, HHSN268201100001C, HHSN268201100002C, HHSN268201100003C, HHSN268201100004C, and HHSN271201100004C.

## Conflict of Interest

The authors declare that the research was conducted in the absence of any commercial or financial relationships that could be construed as a potential conflict of interest.
