# Supplementary material for: Interactions Between Adiponectin-Pathway Polymorphisms and Obesity on Postmenopausal Breast Cancer Risk Among African American Women: The WHI SHARe Study
Source: Front Oncol. 2021 Jul 21;11:698198. doi: 10.3389/fonc.2021.698198 (PMC8335565; doi:10.3389/fonc.2021.698198)
Supplement: Supplementary file 1 [file DataSheet_1.docx]

**Supplementary Figure 1**. A diagram of an interaction effect between *adiponectin-related single nucleotide polymorphisms (SNPs)* and *obesity status* on *invasive breast cancer*.

**Adiponectin-related SNPs**

**(independent variable)**

**Obesity status**

**(effect modifier)**

**SNPs** X **obesity status**

**Invasive Breast Cancer**

**(dependent variable)**

A direct effect (black line) of *adiponectin-related SNPs* is expected on the risk of *invasive breast cancer*. Additionally, both *adiponectin-related SNPs* and *obesity status* that interact and as the *SNPs X obesity status*, influence the effect of *adiponectin-related SNPs* on *invasive breast cancer* (blue lines).
