# Supplementary material for: Interactions Between Adiponectin-Pathway Polymorphisms and Obesity on Postmenopausal Breast Cancer Risk Among African American Women: The WHI SHARe Study
Source: Front Oncol. 2021 Jul 21;11:698198. doi: 10.3389/fonc.2021.698198 (PMC8335565; doi:10.3389/fonc.2021.698198)
Supplement: Supplementary file 2 [file Table_1.docx]

| **Supp Table 1. Allele frequencies of SNPs by invasive breast cancer status** | | | | | | |
| --- | --- | --- | --- | --- | --- | --- |
|  |  |  |  |  | **Effect allele frequency** | |
| **SNP** | **Chromosome** | **Position** | **Associated gene** | **Risk allele (effect/other)** | **Breast cancer (N = 402)** | **No breast cancer (N = 7589)** |
| rs3001167 | 1 | 194530914 | RNU6-983P | C/T | 0.56 | 0.54 |
| rs2232853 | 1 | 202931958 | ADIPOR1 | T/C | 0.15 | 0.15 |
| rs2791553 | 1 | 219676042 | LYPLAL1 | T/C | 0.43 | 0.45 |
| rs4301033 | 3 | 150042618 | EEF1A1P14 | A/G | 0.19 | 0.19 |
| rs822354 | 3 | 186480206 | ADIPOQ | A/G | 0.21 | 0.21 |
| rs266719 | 3 | 186501648 | EIF4A2 | T/C | 0.07 | 0.08 |
| rs864265 | 3 | 186554292 | ADIPOQ | A/C | 0.15 | 0.14 |
| rs182052 | 3 | 186560782 | ADIPOQ | T/C | 0.35 | 0.36 |
| rs17366568 | 3 | 186570453 | ADIPOQ | T/C | 0.53 | 0.03 |
| rs3821799 | 3 | 186571486 | ADIPOQ | C/T | 0.44 | 0.44 |
| rs3774261^a^ | 3 | 186571559 | ADIPOQ | C/T | 0.45 | 0.44 |
| rs6444174^a^ | 3 | 186573189 | ADIPOQ | C/T | 0.04 | 0.15 |
| rs6773957^a^ | 3 | 186573705 | ADIPOQ | C/T | 0.46 | 0.45 |
| rs10517133 | 4 | 45364914 | AC108043.3-1 | C/G | 0.09 | 0.08 |
| rs13434995 | 4 | 56467214 | NMU | G/A | 0.15 | 0.14 |
| rs10012953 | 4 | 110964362 | ELOVL6 | C/T | 0.18 | 0.18 |
| rs13358260 | 5 | 78845711 | PAPD4 | G/A | 0.52 | 0.02 |
| rs10447248 | 5 | 107915736 | FER | T/C | 0.17 | 0.15 |
| rs7722022 | 5 | 172934898 | CTB-164N12.1 | A/C | 0.37 | 0.38 |
| rs4716055 | 6 | 9853919 | OFCC1 | G/T | 0.55 | 0.04 |
| rs998584 | 6 | 43757896 | VEGFA | A/C | 0.23 | 0.22 |
| rs592423 | 6 | 139840693 | RP11-12A2.3 | C/A | 0.4 | 0.41 |
| rs12211360 | 6 | 143119832 | HIVEP2 | G/A | 0.034 | 0.036 |
| rs2468677 | 8 | 140519517 | KCNK9 | C/A | 0.5 | 0.48 |
| rs10746997 | 9 | 78841401 | PCSK5 | C/A | 0.22 | 0.2 |
| rs7128099 | 11 | 20356444 | HTATIP2 | G/A | 0.5 | 0.46 |
| rs7955516 | 12 | 20498036 | LINC02468 | G/T | 0.29 | 0.3 |
| rs11168618 | 12 | 48933233 | OR8S1 | T/C | 0.09 | 0.11 |
| rs2657888 | 12 | 56938383 | RBMS2 | A/C | 0.51 | 0.53 |
| rs10847980 | 12 | 123387922 | VPS37B | G/T | 0.27 | 0.27 |
| rs1187415 | 12 | 124491529 | ZNF664 | C/G | 0.58 | 0.56 |
| rs3865188 | 16 | 82650717 | CDH13 | A/T | 0.4 | 0.38 |

^a^High linkage disequilibrium (r^2^ > 0.80) between all pairs of these three SNPs in *ADIPOQ*.
