# Supplementary material for: Interactions Between Adiponectin-Pathway Polymorphisms and Obesity on Postmenopausal Breast Cancer Risk Among African American Women: The WHI SHARe Study
Source: Front Oncol. 2021 Jul 21;11:698198. doi: 10.3389/fonc.2021.698198 (PMC8335565; doi:10.3389/fonc.2021.698198)
Supplement: Supplementary file 3 [file Table_2.docx]

| **Supp Table 2. Associations of 32 adiponectin-related SNPs and postmenopausal invasive breast cancer risk with or without adjusting for BMI, WHR, and WC** | | | | | | | | | | | | | | | | | |
| --- | --- | --- | --- | --- | --- | --- | --- | --- | --- | --- | --- | --- | --- | --- | --- | --- | --- |
|  |  | **No adjustment for body fat distributions** | | | | **Additional adjustment for BMI** | | | | **Additional adjustment for WHR** | | | | **Additional adjustment for WC** | | | |
| **Genotype** |  | **Model 1^a^** | | **Model 2^b^** | | **Model 1^a^** | | **Model 2^b^** | | **Model 1^a^** |  | **Model 2^b^** |  | **Model 1^a^** | | **Model 2^b^** | |
|  | **brca/no** | **HR (95% CI)** | **p** | **HR (95% CI)** | **p** | **HR (95% CI)** | **p** | **HR (95% CI)** | **p** | **HR (95% CI)** | **p** | **HR (95% CI)** | **p** | **HR (95% CI)** | **p** | **HR (95% CI)** | **p** |
| rs3001167 |  |  |  |  |  |  |  |  |  |  |  |  |  |  |  |  |  |
| TT | 72/1,418 | 1 |  | 1 |  | 1 |  | 1 |  | 1 |  | 1 |  | 1 |  | 1 |  |
| CT | 164/3,372 | 0.97 (0.73, 1.28) | 0.81 | 0.93 (0.69, 1.26) | 0.65 | 0.98 (0.74, 1.30) | 0.91 | 0.95 (0.70, 1.29) | 0.74 | 0.97 (0.73, 1.27) | 0.81 | 0.93 (0.69, 1.26) | 0.65 | 0.97 (0.73, 1.28) | 0.81 | 0.93 (0.69, 1.26) | 0.65 |
| CC | 116/1,946 | 1.19 (0.89, 1.60) | 0.25 | 1.08 (0.78, 1.50) | 0.64 | 1.21 (0.90, 1.63) | 0.21 | 1.10 (0.80, 1.54) | 0.55 | 1.19 (0.89, 1.59) | 0.25 | 1.08 (0.78, 1.50) | 0.63 | 1.19 (0.89, 1.60) | 0.25 | 1.08 (0.78, 1.50) | 0.64 |
| rs2232853 |  |  |  |  |  |  |  |  |  |  |  |  |  |  |  |  |  |
| CC | 253/4,842 | 1 |  | 1 |  | 1 |  | 1 |  | 1 |  | 1 |  | 1 |  | 1 |  |
| TC | 92/1,774 | 0.98 (0.78, 1.25) | 0.89 | 1.03 (0.79, 1.34) | 0.84 | 0.99 (0.78, 1.26) | 0.93 | 1.03 (0.79, 1.35) | 0.81 | 0.99 (0.78, 1.25) | 0.90 | 1.03 (0.79, 1.34) | 0.83 | 0.98 (0.78, 1.25) | 0.89 | 1.03 (0.79, 1.33) | 0.86 |
| TT | 7/147 | 0.92 (0.43, 1.95) | 0.83 | 0.98 (0.44, 2.22) | 0.97 | 0.93 (0.44, 1.98) | 0.85 | 1.00 (0.44, 2.26) | 1.00 | 0.92 (0.43, 1.95) | 0.82 | 0.99 (0.44, 2.22) | 0.97 | 0.92 (0.43, 1.95) | 0.83 | 0.98 (0.44, 2.22) | 0.97 |
| rs2791553 |  |  |  |  |  |  |  |  |  |  |  |  |  |  |  |  |  |
| CC | 106/2,050 | 1 |  | 1 |  | 1 |  | 1 |  | 1 |  | 1 |  | 1 |  | 1 |  |
| TC | 191/3,329 | 1.10 (0.87, 1.40) | 0.42 | 1.02 (0.79, 1.33) | 0.87 | 1.10 (0.87, 1.40) | 0.43 | 1.02 (0.78, 1.33) | 0.88 | 1.11 (0.87, 1.40) | 0.41 | 1.03 (0.79, 1.34) | 0.85 | 1.10 (0.87, 1.40) | 0.42 | 1.02 (0.79, 1.33) | 0.87 |
| TT | 55/1,385 | 0.77 (0.56, 1.07) | 0.11 | 0.79 (0.56, 1.12) | 0.19 | 0.77 (0.55, 1.06) | 0.11 | 0.79 (0.55, 1.12) | 0.19 | 0.77 (0.56, 1.07) | 0.12 | 0.80 (0.56, 1.13) | 0.21 | 0.77 (0.56, 1.07) | 0.11 | 0.79 (0.56, 1.12) | 0.19 |
| rs4301033 |  |  |  |  |  |  |  |  |  |  |  |  |  |  |  |  |  |
| GG | 236/4,455 | 1 |  | 1 |  | 1 |  | 1 |  | 1 |  | 1 |  | 1 |  | 1 |  |
| AG | 99/2,049 | 0.92 (0.73, 1.17) | 0.50 | 0.95 (0.73, 1.23) | 0.70 | 0.92 (0.73, 1.16) | 0.48 | 0.95 (0.73, 1.24) | 0.71 | 0.92 (0.73, 1.16) | 0.49 | 0.95 (0.73, 1.24) | 0.71 | 0.92 (0.73, 1.17) | 0.50 | 0.95 (0.73, 1.23) | 0.70 |
| AA | 17/249 | 1.29 (0.79, 2.11) | 0.32 | 1.52 (0.91, 2.53) | 0.11 | 1.30 (0.79, 2.13) | 0.30 | 1.54 (0.92, 2.57) | 0.10 | 1.29 (0.79, 2.11) | 0.31 | 1.52 (0.91, 2.53) | 0.11 | 1.29 (0.79, 2.11) | 0.32 | 1.52 (0.91, 2.53) | 0.11 |
| rs822354 |  |  |  |  |  |  |  |  |  |  |  |  |  |  |  |  |  |
| GG | 224/4,159 | 1 |  | 1 |  | 1 |  | 1 |  | 1 |  | 1 |  | 1 |  | 1 |  |
| AG | 107/2,281 | 0.88 (0.70, 1.11) | 0.27 | 0.87 (0.67, 1.12) | 0.28 | 0.88 (0.70, 1.11) | 0.28 | 0.87 (0.68, 1.13) | 0.30 | 0.88 (0.70, 1.11) | 0.28 | 0.88 (0.68, 1.13) | 0.31 | 0.88 (0.70, 1.11) | 0.27 | 0.87 (0.67, 1.13) | 0.29 |
| AA | 19/281 | 1.23 (0.77, 1.96) | 0.39 | 1.22 (0.72, 2.06) | 0.47 | 1.24 (0.78, 1.98) | 0.37 | 1.22 (0.72, 2.07) | 0.45 | 1.23 (0.77, 1.97) | 0.39 | 1.21 (0.72, 2.06) | 0.47 | 1.23 (0.77, 1.96) | 0.39 | 1.22 (0.72, 2.06) | 0.47 |
| rs266719 |  |  |  |  |  |  |  |  |  |  |  |  |  |  |  |  |  |
| CC | 306/5,671 | 1 |  | 1 |  | 1 |  | 1 |  | 1 |  | 1 |  | 1 |  | 1 |  |
| TC | 41/1,036 | 0.74 (0.54, 1.03) | 0.07 | **0.65 (0.44, 0.95)** | **0.03** | 0.74 (0.54, 1.03) | 0.07 | **0.65 (0.44, 0.95)** | **0.03** | 0.74 (0.54, 1.03) | 0.07 | **0.65 (0.44, 0.96)** | **0.03** | 0.74 (0.54, 1.03) | 0.07 | 0.65 (0.44, 0.95) | 0.03 |
| TT | 5/54 | 1.65 (0.68, 4.00) | 0.27 | 1.84 (0.68, 4.95) | 0.23 | 1.65 (0.68, 4.00) | 0.27 | 1.83 (0.68, 4.94) | 0.23 | 1.66 (0.69, 4.02) | 0.26 | 1.85 (0.69, 4.98) | 0.22 | 1.65 (0.68, 4.00) | 0.27 | 1.84 (0.68, 4.95) | 0.23 |
| rs864265 |  |  |  |  |  |  |  |  |  |  |  |  |  |  |  |  |  |
| CC | 253/5,041 | 1 |  | 1 |  | 1 |  | 1 |  | 1 |  | 1 |  | 1 |  | 1 |  |
| AC | 89/1,597 | 1.10 (0.86, 1.40) | 0.45 | 1.17 (0.90, 1.52) | 0.25 | 1.09 (0.86, 1.39) | 0.49 | 1.16 (0.89, 1.51) | 0.28 | 1.10 (0.86, 1.40) | 0.44 | 1.17 (0.90, 1.53) | 0.24 | 1.10 (0.86, 1.40) | 0.45 | 1.17 (0.90, 1.53) | 0.25 |
| AA | 10/123 | 1.56 (0.83, 2.93) | 0.17 | 1.56 (0.77, 3.17) | 0.22 | 1.56 (0.83, 2.94) | 0.17 | 1.56 (0.77, 3.17) | 0.22 | 1.56 (0.83, 2.94) | 0.17 | 1.56 (0.77, 3.17) | 0.22 | 1.56 (0.83, 2.93) | 0.17 | 1.55 (0.76, 3.15) | 0.23 |
| rs182052 |  |  |  |  |  |  |  |  |  |  |  |  |  |  |  |  |  |
| CC | 153/2,763 | 1 |  | 1 |  | 1 |  | 1 |  | 1 |  | 1 |  | 1 |  | 1 |  |
| TC | 154/3,101 | 0.91 (0.73, 1.13) | 0.39 | 0.91 (0.71, 1.16) | 0.44 | 0.91 (0.73, 1.14) | 0.41 | 0.91 (0.71, 1.17) | 0.47 | 0.91 (0.72, 1.13) | 0.38 | 0.91 (0.71, 1.16) | 0.43 | 0.91 (0.73, 1.13) | 0.39 | 0.91 (0.71, 1.16) | 0.44 |
| TT | 45/899 | 0.92 (0.66, 1.28) | 0.61 | 0.95 (0.65, 1.37) | 0.78 | 0.92 (0.66, 1.29) | 0.63 | 0.95 (0.66, 1.38) | 0.81 | 0.92 (0.66, 1.28) | 0.61 | 0.95 (0.66, 1.38) | 0.79 | 0.92 (0.66, 1.28) | 0.61 | 0.95 (0.65, 1.37) | 0.77 |
| rs17366568 |  |  |  |  |  |  |  |  |  |  |  |  |  |  |  |  |  |
| CC | 332/6,410 | 1 |  | 1 |  | 1 |  | 1 |  | 1 |  | 1 |  | 1 |  | 1 |  |
| TC | 20/349 | 1.10 (0.70, 1.73) | 0.67 | 1.07 (0.65, 1.78) | 0.79 | 1.11 (0.71, 1.75) | 0.64 | 1.08 (0.65, 1.79) | 0.76 | 1.11 (0.71, 1.74) | 0.66 | 1.06 (0.64, 1.77) | 0.81 | 1.10 (0.70, 1.73) | 0.67 | 1.07 (0.64, 1.77) | 0.80 |
| TT | 0/3 | 0.00 (0.00, Inf) | 0.99 | 0.00 (0.00, Inf) | 0.99 | 0.00 (0.00, Inf) | 0.99 | 0.00 (0.00, Inf) | 0.99 | 0.00 (0.00, Inf) | 0.99 | 0.00 (0.00, Inf) | 0.99 | 0.00 (0.00, Inf) | 0.99 | 0.00 (0.00, Inf) | 0.99 |
| rs3821799 |  |  |  |  |  |  |  |  |  |  |  |  |  |  |  |  |  |
| TT | 112/2,140 | 1 |  | 1 |  | 1 |  | 1 |  | 1 |  | 1 |  | 1 |  | 1 |  |
| CT | 169/3,330 | 0.98 (0.77, 1.24) | 0.84 | 0.89 (0.69, 1.16) | 0.40 | 0.99 (0.78, 1.25) | 0.90 | 0.90 (0.69, 1.18) | 0.46 | 0.98 (0.77, 1.24) | 0.83 | 0.89 (0.68, 1.16) | 0.40 | 0.98 (0.77, 1.24) | 0.84 | 0.89 (0.69, 1.16) | 0.40 |
| CC | 71/1,294 | 1.05 (0.78, 1.42) | 0.74 | 1.01 (0.73, 1.40) | 0.97 | 1.06 (0.79, 1.43) | 0.70 | 1.02 (0.73, 1.41) | 0.92 | 1.05 (0.78, 1.41) | 0.75 | 1.01 (0.73, 1.40) | 0.97 | 1.05 (0.78, 1.42) | 0.74 | 1.01 (0.73, 1.40) | 0.97 |
| rs3774261^c^ |  |  |  |  |  |  |  |  |  |  |  |  |  |  |  |  |  |
| TT | 110/2,123 | 1 |  | 1 |  | 1 |  | 1 |  | 1 |  | 1 |  | 1 |  | 1 |  |
| CT | 166/3,263 | 0.99 (0.78, 1.26) | 0.92 | 0.89 (0.68, 1.16) | 0.38 | 1.00 (0.78, 1.27) | 0.99 | 0.90 (0.69, 1.18) | 0.44 | 0.99 (0.78, 1.26) | 0.92 | 0.89 (0.68, 1.16) | 0.37 | 0.99 (0.78, 1.26) | 0.92 | 0.89 (0.68, 1.16) | 0.37 |
| CC | 76/1,372 | 1.07 (0.80, 1.44) | 0.64 | 1.01 (0.73, 1.40) | 0.94 | 1.08 (0.81, 1.45) | 0.60 | 1.03 (0.74, 1.42) | 0.88 | 1.07 (0.80, 1.43) | 0.65 | 1.01 (0.73, 1.40) | 0.94 | 1.07 (0.80, 1.44) | 0.64 | 1.01 (0.73, 1.40) | 0.94 |
| rs6444174 ^c^ |  |  |  |  |  |  |  |  |  |  |  |  |  |  |  |  |  |
| TT | 249/4,940 | 1 |  | 1 |  | 1 |  | 1 |  | 1 |  | 1 |  | 1 |  | 1 |  |
| CT | 90/1,658 | 1.08 (0.85, 1.37) | 0.54 | 1.15 (0.89, 1.50) | 0.29 | 1.07 (0.84, 1.36) | 0.59 | 1.14 (0.88, 1.49) | 0.33 | 1.08 (0.85, 1.37) | 0.53 | 1.16 (0.89, 1.51) | 0.28 | 1.08 (0.85, 1.37) | 0.54 | 1.16 (0.89, 1.50) | 0.28 |
| CC | 13/164 | 1.54 (0.88, 2.68) | 0.13 | 1.55 (0.82, 2.92) | 0.18 | 1.54 (0.88, 2.69) | 0.13 | 1.55 (0.82, 2.93) | 0.18 | 1.54 (0.88, 2.69) | 0.13 | 1.56 (0.82, 2.94) | 0.17 | 1.54 (0.88, 2.68) | 0.13 | 1.54 (0.81, 2.91) | 0.19 |
| rs6773957 ^c^ |  |  |  |  |  |  |  |  |  |  |  |  |  |  |  |  |  |
| TT | 104/2,034 | 1 |  | 1 |  | 1 |  | 1 |  | 1 |  | 1 |  | 1 |  | 1 |  |
| CT | 171/3,349 | 1.00 (0.79, 1.28) | 0.98 | 0.92 (0.71, 1.21) | 0.56 | 1.02 (0.80, 1.30) | 0.90 | 0.94 (0.72, 1.23) | 0.64 | 1.00 (0.79, 1.28) | 0.97 | 0.92 (0.70, 1.21) | 0.55 | 1.00 (0.79, 1.28) | 0.98 | 0.92 (0.71, 1.21) | 0.56 |
| CC | 77/1,379 | 1.09 (0.82, 1.47) | 0.55 | 1.03 (0.75, 1.43) | 0.85 | 1.10 (0.82, 1.48) | 0.51 | 1.05 (0.76, 1.45) | 0.79 | 1.09 (0.81, 1.47) | 0.56 | 1.03 (0.75, 1.43) | 0.85 | 1.09 (0.82, 1.47) | 0.55 | 1.03 (0.75, 1.43) | 0.85 |
| rs10517133 |  |  |  |  |  |  |  |  |  |  |  |  |  |  |  |  |  |
| GG | 293/5,679 | 1 |  | 1 |  | 1 |  | 1 |  | 1 |  | 1 |  | 1 |  | 1 |  |
| CG | 56/1,027 | 1.06 (0.79, 1.41) | 0.70 | 1.05 (0.76, 1.44) | 0.77 | 1.06 (0.80, 1.41) | 0.69 | 1.05 (0.77, 1.45) | 0.75 | 1.06 (0.79, 1.40) | 0.72 | 1.04 (0.76, 1.43) | 0.80 | 1.06 (0.79, 1.41) | 0.70 | 1.05 (0.76, 1.44) | 0.78 |
| CC | 3/52 | 1.11 (0.36, 3.47) | 0.85 | 0.89 (0.22, 3.59) | 0.87 | 1.11 (0.36, 3.45) | 0.86 | 0.89 (0.22, 3.57) | 0.86 | 1.13 (0.36, 3.52) | 0.84 | 0.92 (0.23, 3.69) | 0.90 | 1.11 (0.36, 3.47) | 0.85 | 0.89 (0.22, 3.59) | 0.87 |
| rs13434995 |  |  |  |  |  |  |  |  |  |  |  |  |  |  |  |  |  |
| AA | 253/5,032 | 1 |  | 1 |  | 1 |  | 1 |  | 1 |  | 1 |  | 1 |  | 1 |  |
| GA | 91/1,617 | 1.11 (0.88, 1.42) | 0.38 | 1.20 (0.92, 1.56) | 0.19 | 1.12 (0.88, 1.43) | 0.35 | 1.21 (0.93, 1.57) | 0.17 | 1.12 (0.88, 1.42) | 0.37 | 1.21 (0.93, 1.57) | 0.17 | 1.11 (0.88, 1.42) | 0.38 | 1.20 (0.92, 1.56) | 0.18 |
| GG | 8/115 | 1.35 (0.67, 2.74) | 0.40 | 1.83 (0.90, 3.71) | 0.10 | 1.37 (0.68, 2.77) | 0.38 | 1.86 (0.92, 3.78) | 0.09 | 1.35 (0.67, 2.73) | 0.40 | 1.82 (0.90, 3.70) | 0.10 | 1.35 (0.67, 2.74) | 0.40 | 1.82 (0.90, 3.70) | 0.10 |
| rs10012953 |  |  |  |  |  |  |  |  |  |  |  |  |  |  |  |  |  |
| TT | 238/4,540 | 1 |  | 1 |  | 1 |  | 1 |  | 1 |  | 1 |  | 1 |  | 1 |  |
| CT | 100/1,987 | 0.96 (0.76, 1.21) | 0.72 | 1.08 (0.84, 1.39) | 0.56 | 0.96 (0.76, 1.21) | 0.74 | 1.08 (0.84, 1.40) | 0.54 | 0.96 (0.76, 1.21) | 0.72 | 1.08 (0.83, 1.39) | 0.57 | 0.96 (0.76, 1.21) | 0.72 | 1.08 (0.84, 1.40) | 0.56 |
| CC | 13/230 | 1.08 (0.62, 1.89) | 0.78 | 1.42 (0.81, 2.49) | 0.23 | 1.08 (0.62, 1.89) | 0.78 | 1.42 (0.81, 2.49) | 0.23 | 1.09 (0.62, 1.90) | 0.77 | 1.43 (0.81, 2.51) | 0.21 | 1.08 (0.62, 1.89) | 0.78 | 1.42 (0.81, 2.50) | 0.22 |
| rs13358260 |  |  |  |  |  |  |  |  |  |  |  |  |  |  |  |  |  |
| AA | 338/6,525 | 1 |  | 1 |  | 1 |  | 1 |  | 1 |  | 1 |  | 1 |  | 1 |  |
| GA | 14/233 | 1.15 (0.67, 1.96) | 0.61 | 1.12 (0.63, 2.01) | 0.69 | 1.15 (0.68, 1.97) | 0.60 | 1.13 (0.64, 2.02) | 0.67 | 1.16 (0.68, 1.97) | 0.60 | 1.14 (0.64, 2.03) | 0.67 | 1.15 (0.67, 1.96) | 0.61 | 1.13 (0.63, 2.02) | 0.68 |
| GG | 0/5 | 0.00 (0.00, Inf) | 0.99 | 0.00 (0.00, Inf) | 0.99 | 0.00 (0.00, Inf) | 0.99 | 0.00 (0.00, Inf) | 0.99 | 0.00 (0.00, Inf) | 0.99 | 0.00 (0.00, Inf) | 0.99 | 0.00 (0.00, Inf) | 0.99 | 0.00 (0.00, Inf) | 0.99 |
| rs10447248 |  |  |  |  |  |  |  |  |  |  |  |  |  |  |  |  |  |
| CC | 245/4,881 | 1 |  | 1 |  | 1 |  | 1 |  | 1 |  | 1 |  | 1 |  | 1 |  |
| TC | 94/1,728 | 1.08 (0.85, 1.37) | 0.53 | 1.15 (0.88, 1.49) | 0.31 | 1.07 (0.84, 1.36) | 0.59 | 1.13 (0.87, 1.47) | 0.35 | 1.08 (0.85, 1.37) | 0.53 | 1.15 (0.89, 1.50) | 0.29 | 1.08 (0.85, 1.37) | 0.53 | 1.15 (0.88, 1.49) | 0.30 |
| TT | 13/152 | 1.64 (0.94, 2.87) | 0.08 | 1.56 (0.83, 2.96) | 0.17 | 1.65 (0.95, 2.88) | 0.08 | 1.58 (0.83, 2.98) | 0.16 | 1.63 (0.93, 2.85) | 0.09 | 1.55 (0.82, 2.94) | 0.18 | 1.64 (0.94, 2.87) | 0.08 | 1.57 (0.83, 2.97) | 0.17 |
| rs7722022 |  |  |  |  |  |  |  |  |  |  |  |  |  |  |  |  |  |
| CC | 145/2,567 | 1 |  | 1 |  | 1 |  | 1 |  | 1 |  | 1 |  | 1 |  | 1 |  |
| AC | 157/3,229 | 0.86 (0.69, 1.08) | 0.20 | 0.88 (0.68, 1.13) | 0.30 | 0.86 (0.69, 1.08) | 0.20 | 0.88 (0.68, 1.13) | 0.30 | 0.86 (0.69, 1.08) | 0.20 | 0.87 (0.68, 1.13) | 0.30 | 0.86 (0.69, 1.08) | 0.20 | 0.88 (0.68, 1.13) | 0.31 |
| AA | 50/966 | 0.90 (0.65, 1.25) | 0.53 | 1.00 (0.70, 1.41) | 0.98 | 0.88 (0.64, 1.22) | 0.45 | 0.97 (0.68, 1.38) | 0.86 | 0.90 (0.65, 1.25) | 0.53 | 0.99 (0.70, 1.41) | 0.96 | 0.90 (0.65, 1.25) | 0.53 | 0.99 (0.70, 1.41) | 0.97 |
| rs4716055 |  |  |  |  |  |  |  |  |  |  |  |  |  |  |  |  |  |
| TT | 319/6,208 | 1 |  | 1 |  | 1 |  | 1 |  | 1 |  | 1 |  | 1 |  | 1 |  |
| GT | 33/540 | 1.17 (0.82, 1.67) | 0.40 | 1.16 (0.78, 1.72) | 0.47 | 1.17 (0.82, 1.68) | 0.39 | 1.16 (0.78, 1.73) | 0.46 | 1.16 (0.81, 1.66) | 0.42 | 1.15 (0.77, 1.71) | 0.50 | 1.17 (0.82, 1.67) | 0.40 | 1.16 (0.78, 1.72) | 0.48 |
| GG | 0/15 | 0.00 (0.00, Inf) | 0.99 | 0.00 (0.00, Inf) | 0.99 | 0.00 (0.00, Inf) | 0.99 | 0.00 (0.00, Inf) | 0.99 | 0.00 (0.00, Inf) | 0.99 | 0.00 (0.00, Inf) | 0.99 | 0.00 (0.00, Inf) | 0.99 | 0.00 (0.00, Inf) | 0.99 |
| rs998584 |  |  |  |  |  |  |  |  |  |  |  |  |  |  |  |  |  |
| CC | 205/4,106 | 1 |  | 1 |  | 1 |  | 1 |  | 1 |  | 1 |  | 1 |  | 1 |  |
| AC | 129/2,313 | 1.11 (0.89, 1.38) | 0.36 | 1.19 (0.93, 1.52) | 0.16 | 1.10 (0.88, 1.37) | 0.42 | 1.18 (0.92, 1.51) | 0.19 | 1.11 (0.89, 1.38) | 0.37 | 1.19 (0.93, 1.52) | 0.17 | 1.11 (0.89, 1.38) | 0.36 | 1.19 (0.94, 1.52) | 0.16 |
| AA | 18/344 | 1.02 (0.63, 1.66) | 0.92 | 1.24 (0.75, 2.05) | 0.40 | 1.02 (0.63, 1.65) | 0.94 | 1.23 (0.75, 2.03) | 0.42 | 1.02 (0.63, 1.65) | 0.94 | 1.22 (0.74, 2.02) | 0.43 | 1.02 (0.63, 1.66) | 0.92 | 1.24 (0.75, 2.04) | 0.41 |
| rs592423 |  |  |  |  |  |  |  |  |  |  |  |  |  |  |  |  |  |
| AA | 124/2,310 | 1 |  | 1 |  | 1 |  | 1 |  | 1 |  | 1 |  | 1 |  | 1 |  |
| CA | 176/3,355 | 0.99 (0.78, 1.24) | 0.91 | 1.07 (0.83, 1.38) | 0.61 | 0.98 (0.78, 1.23) | 0.86 | 1.06 (0.82, 1.37) | 0.65 | 0.98 (0.78, 1.24) | 0.88 | 1.07 (0.82, 1.38) | 0.63 | 0.99 (0.78, 1.24) | 0.91 | 1.07 (0.83, 1.38) | 0.61 |
| CC | 52/1,099 | 0.89 (0.64, 1.23) | 0.48 | 0.89 (0.62, 1.29) | 0.54 | 0.89 (0.65, 1.23) | 0.49 | 0.89 (0.62, 1.29) | 0.54 | 0.89 (0.64, 1.23) | 0.47 | 0.89 (0.62, 1.28) | 0.52 | 0.89 (0.64, 1.23) | 0.48 | 0.89 (0.62, 1.28) | 0.54 |
| rs12211360 |  |  |  |  |  |  |  |  |  |  |  |  |  |  |  |  |  |
| AA | 329/6,285 | 1 |  | 1 |  | 1 |  | 1 |  | 1 |  | 1 |  | 1 |  | 1 |  |
| GA | 22/469 | 0.88 (0.57, 1.36) | 0.57 | 0.90 (0.56, 1.45) | 0.67 | 0.89 (0.58, 1.36) | 0.58 | 0.90 (0.56, 1.45) | 0.66 | 0.88 (0.57, 1.36) | 0.57 | 0.89 (0.55, 1.44) | 0.64 | 0.88 (0.57, 1.36) | 0.57 | 0.90 (0.56, 1.45) | 0.66 |
| GG | 1/10 | 1.73 (0.24, 12.29) | 0.59 | 2.04 (0.29, 14.59) | 0.48 | 1.77 (0.25, 12.61) | 0.57 | 2.08 (0.29, 14.93) | 0.47 | 1.71 (0.24, 12.15) | 0.59 | 2.01 (0.28, 14.40) | 0.49 | 1.73 (0.24, 12.29) | 0.59 | 2.03 (0.28, 14.51) | 0.48 |
| rs2468677 |  |  |  |  |  |  |  |  |  |  |  |  |  |  |  |  |  |
| AA | 81/1,841 | 1 |  | 1 |  | 1 |  | 1 |  | 1 |  | 1 |  | 1 |  | 1 |  |
| CA | 188/3,353 | 1.27 (0.98, 1.64) | 0.08 | 1.33 (0.99, 1.77) | 0.06 | 1.28 (0.99, 1.66) | 0.07 | **1.35 (1.01, 1.80)** | **<0.05** | 1.27 (0.98, 1.64) | 0.08 | 1.33 (0.99, 1.77) | 0.06 | 1.27 (0.98, 1.64) | 0.08 | 1.33 (1.00, 1.77) | 0.05 |
| CC | 83/1,569 | 1.20 (0.88, 1.63) | 0.25 | 1.14 (0.80, 1.61) | 0.47 | 1.21 (0.89, 1.65) | 0.22 | 1.15 (0.81, 1.64) | 0.43 | 1.20 (0.89, 1.63) | 0.24 | 1.14 (0.81, 1.62) | 0.45 | 1.20 (0.88, 1.63) | 0.25 | 1.14 (0.80, 1.61) | 0.47 |
| rs10746997 |  |  |  |  |  |  |  |  |  |  |  |  |  |  |  |  |  |
| AA | 213/4,309 | 1 |  | 1 |  | 1 |  | 1 |  | 1 |  | 1 |  | 1 |  | 1 |  |
| CA | 125/2,182 | 1.16 (0.93, 1.45) | 0.19 | 1.09 (0.85, 1.39) | 0.51 | 1.17 (0.94, 1.46) | 0.17 | 1.10 (0.86, 1.41) | 0.47 | 1.16 (0.93, 1.44) | 0.20 | 1.08 (0.85, 1.39) | 0.52 | 1.16 (0.93, 1.45) | 0.19 | 1.09 (0.85, 1.39) | 0.51 |
| CC | 14/273 | 1.02 (0.60, 1.76) | 0.93 | 1.05 (0.58, 1.88) | 0.87 | 1.04 (0.60, 1.78) | 0.90 | 1.06 (0.59, 1.91) | 0.84 | 1.02 (0.60, 1.76) | 0.94 | 1.05 (0.58, 1.88) | 0.88 | 1.02 (0.60, 1.76) | 0.93 | 1.05 (0.58, 1.88) | 0.88 |
| rs7128099 |  |  |  |  |  |  |  |  |  |  |  |  |  |  |  |  |  |
| AA | 88/1,934 | 1 |  | 1 |  | 1 |  | 1 |  | 1 |  | 1 |  | 1 |  | 1 |  |
| GA | 179/3,400 | 1.16 (0.90, 1.50) | 0.26 | 1.23 (0.92, 1.63) | 0.16 | 1.15 (0.89, 1.48) | 0.29 | 1.22 (0.92, 1.62) | 0.17 | 1.16 (0.90, 1.49) | 0.26 | 1.23 (0.93, 1.64) | 0.15 | 1.16 (0.90, 1.50) | 0.26 | 1.23 (0.93, 1.64) | 0.15 |
| GG | 85/1,429 | 1.32 (0.98, 1.77) | 0.07 | 1.35 (0.97, 1.88) | 0.08 | 1.31 (0.97, 1.77) | 0.08 | 1.35 (0.96, 1.88) | 0.08 | 1.32 (0.98, 1.78) | 0.07 | 1.36 (0.97, 1.90) | 0.07 | 1.32 (0.98, 1.77) | 0.07 | 1.35 (0.97, 1.89) | 0.08 |
| rs7955516 |  |  |  |  |  |  |  |  |  |  |  |  |  |  |  |  |  |
| TT | 176/3,323 | 1 |  | 1 |  | 1 |  | 1 |  | 1 |  | 1 |  | 1 |  | 1 |  |
| GT | 149/2,845 | 0.99 (0.80, 1.23) | 0.92 | 0.93 (0.73, 1.18) | 0.54 | 1.00 (0.80, 1.24) | 0.97 | 0.93 (0.73, 1.19) | 0.58 | 0.99 (0.79, 1.23) | 0.91 | 0.93 (0.73, 1.18) | 0.54 | 0.99 (0.80, 1.23) | 0.92 | 0.93 (0.73, 1.18) | 0.54 |
| GG | 27/593 | 0.87 (0.58, 1.30) | 0.50 | 0.96 (0.63, 1.48) | 0.86 | 0.88 (0.59, 1.32) | 0.53 | 0.97 (0.63, 1.50) | 0.89 | 0.87 (0.58, 1.30) | 0.50 | 0.96 (0.62, 1.48) | 0.86 | 0.87 (0.58, 1.30) | 0.50 | 0.96 (0.62, 1.48) | 0.86 |
| rs11168618 |  |  |  |  |  |  |  |  |  |  |  |  |  |  |  |  |  |
| CC | 297/5,324 | 1 |  | 1 |  | 1 |  | 1 |  | 1 |  | 1 |  | 1 |  | 1 |  |
| TC | 49/1,338 | **0.65 (0.48, 0.88)** | **0.01** | 0.73 (0.53, 1.01) | 0.05 | **0.65 (0.48, 0.88)** | **0.01** | 0.74 (0.53, 1.02) | 0.06 | **0.65 (0.48, 0.87)** | **< 0.01** | **0.72 (0.52, 1.00)** | **<0.05** | **0.65 (0.48, 0.88)** | **0.01** | **0.73 (0.53, 1.01)** | 0.05 |
| TT | 6/102 | 1.02 (0.46, 2.30) | 0.96 | 1.08 (0.45, 2.63) | 0.86 | 1.01 (0.45, 2.27) | 0.98 | 1.08 (0.45, 2.63) | 0.86 | 1.02 (0.46, 2.29) | 0.96 | 1.09 (0.45, 2.65) | 0.85 | 1.02 (0.46, 2.30) | 0.96 | 1.09 (0.45, 2.66) | 0.84 |
| rs2657888 |  |  |  |  |  |  |  |  |  |  |  |  |  |  |  |  |  |
| CC | 73/1,541 | 1 |  | 1 |  | 1 |  | 1 |  | 1 |  | 1 |  | 1 |  | 1 |  |
| AC | 192/3,248 | 1.25 (0.95, 1.63) | 0.11 | 1.24 (0.92, 1.68) | 0.16 | 1.24 (0.95, 1.62) | 0.12 | 1.23 (0.91, 1.66) | 0.17 | 1.25 (0.95, 1.63) | 0.11 | 1.24 (0.92, 1.68) | 0.15 | 1.25 (0.95, 1.63) | 0.11 | 1.24 (0.92, 1.68) | 0.16 |
| AA | 81/1,881 | 0.92 (0.67, 1.26) | 0.58 | 0.92 (0.65, 1.30) | 0.62 | 0.92 (0.67, 1.26) | 0.60 | 0.92 (0.65, 1.31) | 0.65 | 0.92 (0.67, 1.26) | 0.59 | 0.92 (0.65, 1.30) | 0.63 | 0.92 (0.67, 1.26) | 0.58 | 0.92 (0.65, 1.30) | 0.62 |
| rs10847980 |  |  |  |  |  |  |  |  |  |  |  |  |  |  |  |  |  |
| TT | 192/3,629 | 1 |  | 1 |  | 1 |  | 1 |  | 1 |  | 1 |  | 1 |  | 1 |  |
| GT | 130/2,635 | 0.95 (0.76, 1.18) | 0.63 | 0.88 (0.68, 1.12) | 0.30 | 0.94 (0.76, 1.18) | 0.60 | 0.87 (0.68, 1.12) | 0.28 | 0.95 (0.76, 1.19) | 0.64 | 0.88 (0.68, 1.13) | 0.31 | 0.95 (0.76, 1.18) | 0.63 | 0.88 (0.68, 1.13) | 0.30 |
| GG | 30/498 | 1.16 (0.79, 1.70) | 0.46 | 1.13 (0.73, 1.73) | 0.59 | 1.12 (0.76, 1.66) | 0.56 | 1.08 (0.70, 1.68) | 0.72 | 1.15 (0.79, 1.70) | 0.47 | 1.12 (0.73, 1.73) | 0.60 | 1.16 (0.79, 1.70) | 0.46 | 1.13 (0.73, 1.73) | 0.59 |
| rs1187415 |  |  |  |  |  |  |  |  |  |  |  |  |  |  |  |  |  |
| GG | 62/1,320 | 1 |  | 1 |  | 1 |  | 1 |  | 1 |  | 1 |  | 1 |  | 1 |  |
| CG | 175/3,280 | 1.13 (0.85, 1.51) | 0.41 | 1.04 (0.76, 1.43) | 0.80 | 1.13 (0.84, 1.50) | 0.43 | 1.04 (0.76, 1.42) | 0.82 | 1.13 (0.85, 1.51) | 0.40 | 1.04 (0.76, 1.43) | 0.79 | 1.13 (0.85, 1.51) | 0.41 | 1.04 (0.76, 1.43) | 0.80 |
| CC | 115/2,163 | 1.14 (0.84, 1.55) | 0.42 | 1.06 (0.76, 1.49) | 0.72 | 1.13 (0.83, 1.54) | 0.45 | 1.05 (0.75, 1.47) | 0.77 | 1.14 (0.84, 1.55) | 0.41 | 1.07 (0.77, 1.50) | 0.69 | 1.14 (0.84, 1.55) | 0.42 | 1.07 (0.76, 1.49) | 0.70 |
| rs3865188 |  |  |  |  |  |  |  |  |  |  |  |  |  |  |  |  |  |
| TT | 123/2,644 | 1 |  | 1 |  | 1 |  | 1 |  | 1 |  | 1 |  | 1 |  | 1 |  |
| AT | 175/3,149 | 1.20 (0.95, 1.51) | 0.12 | 1.19 (0.92, 1.53) | 0.19 | 1.19 (0.95, 1.50) | 0.14 | 1.18 (0.92, 1.53) | 0.20 | 1.20 (0.95, 1.51) | 0.12 | 1.19 (0.92, 1.54) | 0.18 | 1.20 (0.95, 1.51) | 0.12 | 1.19 (0.92, 1.54) | 0.18 |
| AA | 54/971 | 1.20 (0.87, 1.65) | 0.27 | 1.19 (0.83, 1.70) | 0.35 | 1.20 (0.87, 1.65) | 0.27 | 1.19 (0.83, 1.70) | 0.35 | 1.20 (0.87, 1.65) | 0.27 | 1.20 (0.84, 1.71) | 0.33 | 1.20 (0.87, 1.65) | 0.27 | 1.19 (0.83, 1.70) | 0.35 |

Note: Boldface text indicates statistical significance at *P* < 0.05.

Abbreviation: Chr, chromosome; brca, invasive breast cancer; BMI, body mass index; WHR, waist-to-hip ratio; WC, waist circumference; CI, confidence interval

^a^Adjusted for age only.

^b^Adjusted for age, dietary alcohol (g), diabetes, dietary fat (g), depression scale, energy expenditure, employment status, ever smoking status, number of pregnancies, age at menopause, income status, unopposed estrogen use ever, unopposed estrogen + progesterone use ever.

^c^High linkage disequilibrium (r^2^ > 0.80) was found between all pairs of these three SNPs in *ADIPOQ*.
